# FGF-2 signaling in nasopharyngeal carcinoma modulates pericyte-macrophage crosstalk and metastasis

**DOI:** 10.1172/jci.insight.157874

**Published:** 2022-05-23

**Authors:** Yujie Wang, Qi Sun, Ying Ye, Xiaoting Sun, Sisi Xie, Yuhang Zhan, Jian Song, Xiaoqin Fan, Bin Zhang, Ming Yang, Lei Lv, Kayoko Hosaka, Yunlong Yang, Guohui Nie

**Affiliations:** 1 Shenzhen Key Laboratory of Nanozymes and Translational Cancer Research, Department of Otolaryngology, Shenzhen Institute of Translational Medicine, The First Affiliated Hospital of Shenzhen University, Shenzhen Second People’s Hospital, Shenzhen, Guangdong, China.; 2 Department of Cellular and Genetic Medicine, School of Basic Medical Sciences, Fudan University, Shanghai, China.; 3 Department of Oral Implantology, School and Hospital of Stomatology, Tongji University, Shanghai Engineering Research Center of Tooth Restoration and Regeneration, Shanghai, China.; 4 Department of Microbiology, Tumor and Cell Biology, Karolinska Institutet, Stockholm, Sweden.; 5 Department of Otolaryngology, Shenzhen People’s Hospital, the Second Clinical Medical College, Jinan University, the First Affiliated Hospital, Southern University of Science and Technology, Shenzhen, Guangdong, China.; 6 Ministry of Education Key Laboratory of Metabolism and Molecular Medicine, Department of Biochemistry and Molecular Biology, School of Basic Medical Sciences, Fudan University, Shanghai, China.; 7 State Key Laboratory of Chemical Oncogenomics, Guangdong Provincial Key Laboratory of Chemical Genomics, Peking University Shenzhen Graduate School, Shenzhen, Guangdong, China.

**Keywords:** Immunology, Oncology, Cancer, Macrophages, Pericytes

## Abstract

Molecular signaling in the tumor microenvironment (TME) is complex, and crosstalk among various cell compartments in supporting metastasis remains poorly understood. In particular, the role of vascular pericytes, a critical cellular component in the TME, in cancer invasion and metastasis warrants further investigation. Here, we report that an elevation of FGF-2 signaling in samples from patients with nasopharyngeal carcinoma (NPC) and xenograft mouse models promoted NPC metastasis. Mechanistically, tumor cell–derived FGF-2 strongly promoted pericyte proliferation and pericyte-specific expression of an orphan chemokine (C-X-C motif) ligand 14 (CXCL14) via FGFR1/AHR signaling. Gain- and loss-of-function experiments validated that pericyte-derived CXCL14 promoted macrophage recruitment and polarization toward an M2-like phenotype. Genetic knockdown of *FGF2* or genetic depletion of tumoral pericytes blocked CXCL14 expression and tumor-associated macrophage (TAM) infiltration. Pharmacological inhibition of TAMs by clodronate liposome treatment resulted in a reduction of FGF-2–induced pulmonary metastasis. Together, these findings shed light on the inflammatory role of tumoral pericytes in promoting TAM-mediated metastasis. We provide mechanistic insight into an FGF-2/FGFR1/pericyte/CXCL14/TAM stromal communication axis in NPC and propose an effective antimetastasis therapy concept by targeting a pericyte-derived inflammation for NPC or FGF-2^hi^ tumors.

## Graphical abstract

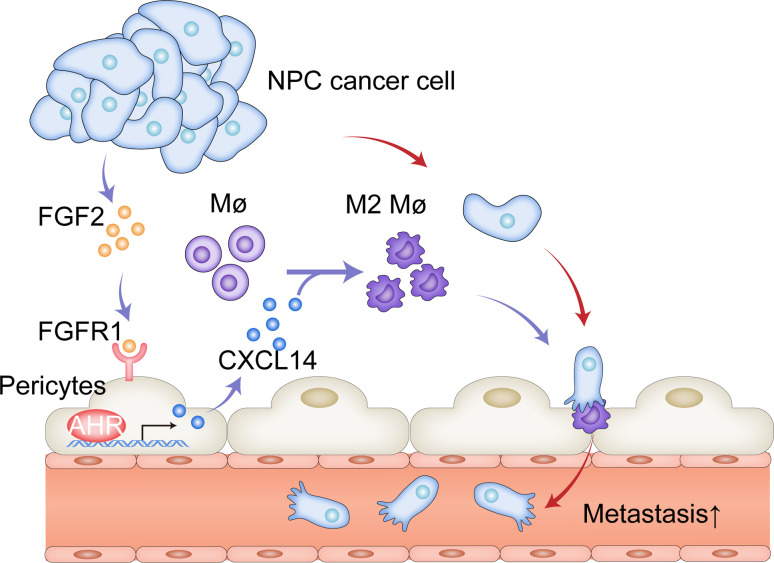

## Introduction

Nasopharyngeal carcinoma (NPC) accounts for 73,000 deaths in 2018, and Southeast Asia exhibits the highest incidence (1, 2). Commonly contributing factors in NPC development include Epstein-Barr virus (EBV) infection, genetic susceptibility, and lifestyle (2). Clinically, radiotherapy and chemotherapy are recommended for early-stage NPC and nonmetastatic NPC patients (3). However, therapeutic options for metastatic NPC patients are limited. Metastatic NPC appears to be a heterogeneous group of tumors with a wide range of survival, and lung, liver, and bone are the most common sites of distant metastases (4). Targeted therapy is recognized as an effective approach to further prolong the survival of NPC patients. Nevertheless, several clinical trials show that targeting the vascular endothelial growth factor (VEGF) signaling by bevacizumab, or targeting the epidermal growth factor (EGF) signaling by cetuximab, did not show clinical benefits in NPC patients, compared with conventional chemoradiotherapy (5–7). Hence, novel molecular-targeted therapies for NPC are urgently warranted. The mechanistic study of NPC metastasis is the foundation of developing novel targeted therapies. Currently, NPC metastasis studies are mostly limited to cancer cells, per se (8). The crosstalk among various cell compartments in the NPC microenvironment are largely overlooked.

Cancer metastasis involves sophisticated interactions between malignant and host cells (9–13). Cancer cells often produce signaling molecules to manipulate host cells to facilitate their invasion and metastasis. Many of these host cells comprise recruited inflammatory cells from circulation. Indeed, cancer tissues often contain an exceptionally high number of inflammatory cells, which significantly promotes cancer metastasis (14). Various inflammatory cytokines and chemokines are involved in recruitment, activation, and polarization of inflammatory cells in the malignancy (15). Interestingly, the recruitment of inflammatory cells is specific to tumor type. For example, pancreatic ductal adenocarcinoma specifically produces a high level of IL-33 explicitly to recruit tumor-associated macrophages (TAMs; ref. 16). However, in NPC, a tumor type containing an exceptionally high number of inflammatory cells (17), intensive research on malignant cell-inflammatory cell interactions has not been conducted.

The fibroblast growth factor–fibroblast growth factor receptor (FGF/FGFR) signaling affects the growth and differentiation of various cell types (18) and often becomes activated in the tumor microenvironment (TME; ref. 19). Of note, various studies have emphasized that the FGF/FGFR signaling triggers interaction between tumor and stromal cells (20). In the TME, FGF primarily targets cells originating from the mesoderm, such as stromal fibroblasts, pericytes, and vascular smooth muscle cells (20). Although it is well known that FGF modulates pericytes for angiogenesis and vascular remodeling (21), its role in orchestrating tumor inflammation is not well understood.

The orphan chemokine (C-X-C motif) ligand 14 (CXCL14) promotes migration of various inflammatory cells and is highly conserved in vertebrates (22). In the tumor, CXCL14 can be expressed by inflammatory cells, fibroblasts, and endothelial cells, and it stimulates its biological activities on endothelial cells, NK cells, neutrophils, DCs, and macrophages (23). In TME, the molecular mechanisms regulating CXCL14 expression are far from clear, and the functional role of tumoral CXCL14 is rather contradictive (24). CXCL14 can regulate calcium influx, NF-κB activity, AP-1 activation, and NOS1 expression as its intracellular molecular targets (23). However, its receptor is poorly defined. A recent study suggested that ACKR2, an atypical G-protein–coupled receptor, mediates CXCL14 function in the epithelial-to-mesenchymal transition (24). The role of CXCL14 in regulating tumor metastasis is not elucidated, and further investigation is warranted in the expanding field of chemokine research in TME.

In this work, we took a cross-platform approach to identify that FGF-2 is highly expressed in clinical NPC samples, and we developed various mouse models to study the molecular mechanisms underlying FGF-2–promoted tumor metastasis. NPC cell–derived FGF-2 strongly promotes vascular-associated pericytes proliferation and expression of CXCL14, which mediates the recruitment and polarization of TAMs and TAM-dependent metastasis. Genetic and pharmacological targeting the FGF-2/FGFR1 signaling or depletion of pericytes obliterates tumoral CXCL14 expression in FGF-2–expressing tumor–bearing mice. TAM depletion significantly reduced FGF-2–mediated NPC metastasis. Thus, targeting the FGF-2/FGFR1/pericyte/CXCL14/TAMs axis provides a potentially novel and rational approach for treating NPC and other FGF-2–expressing tumors. These findings demonstrate a causal link between FGF-2 and CXCL14 for the first time to our knowledge and propose a concept that targeting pericyte-mediated inflammation may serve as an antimetastasis therapy.

## Results

### FGF-2 expression and TAMs infiltration in human NPC tissues.

**Figure 1:**
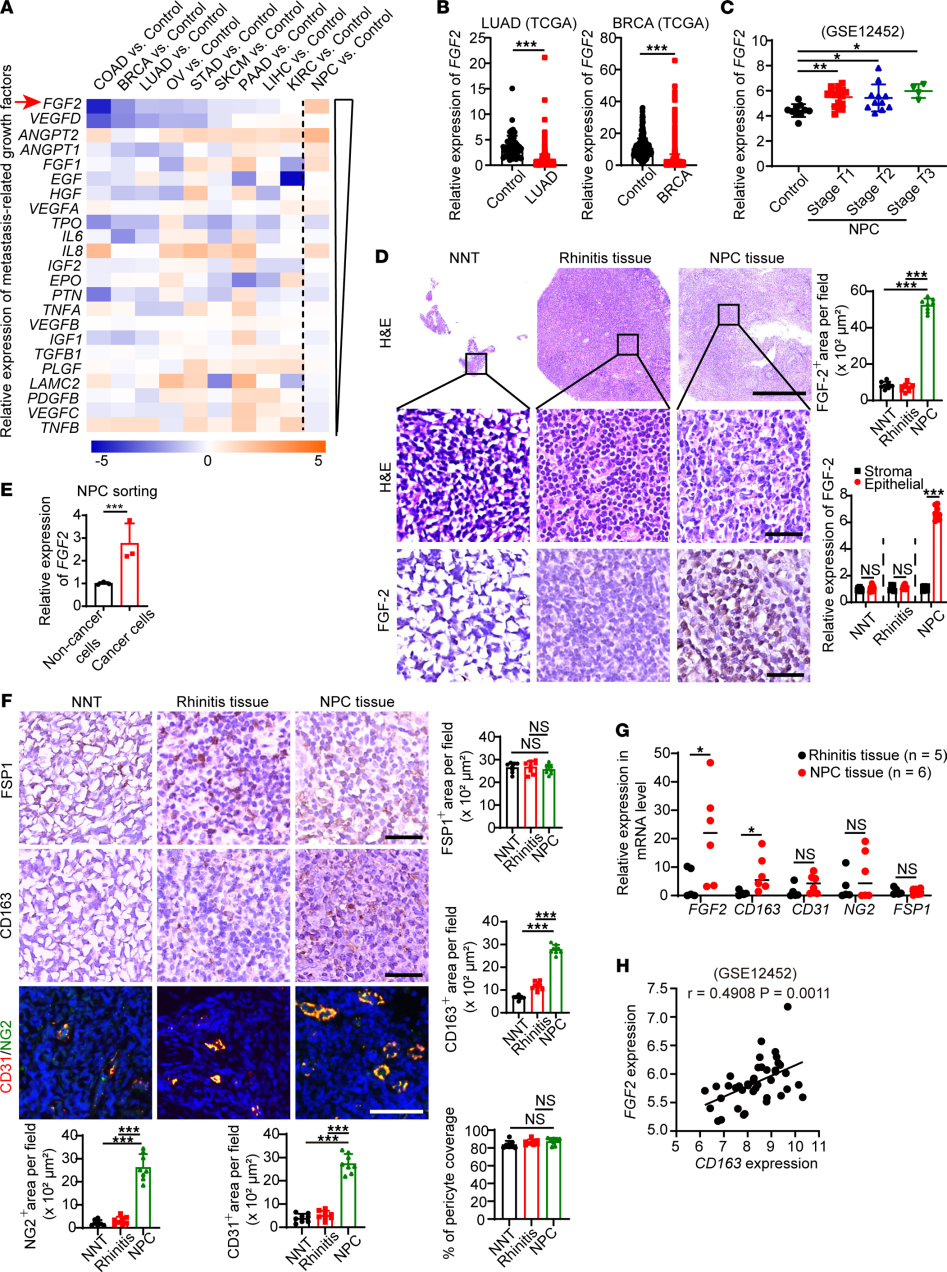
FGF-2 is distinctively expressed and correlates with TAM infiltration in human NPC. (**A**) Cross–data set quantitative heatmap of selected genes of various types of cancer and their adjacent control healthy tissues. Arrow points to distinctively upregulated genes in NPC. Log_2_ fold changes were used for quantification. (**B**) Transcriptomic expression levels of *FGF2* in human LUAD tissues, BRCA tissues and their adjacent healthy tissues. Sample number: control-LUAD/LUAD/control-BRCA/BRCA=347/483/291/1085. (**C**) Transcriptomic expression levels of *FGF2* in various stages of human NPC tissues and their adjacent healthy tissues. Sample number: control/StageT1/StageT2/StageT3=10/16/11/4. (**D**) Human normal nasopharyngeal tissues (NNT), rhinitis tissues, and NPC tissues were stained with H&E and an anti–FGF-2 antibody (brown). Sample number: NNT/Rhinitis/NPC=3/10/6. Scale bar in upper panel: 500 μm. Scale bar in middle and lower panels: 50 μm. Quantification of FGF-2^+^ signals and FGF-2^+^ signals in stromal and epithelial components (*n =* 8 random fields per group). (**E**) NPC cancer cells were sorted by MACS from freshly tissues. qPCR quantification of *FGF2* mRNA (*n =* 3 samples per group). (**F**) NNT rhinitis tissues and NPC tissues were stained. Sample number: NNT/Rhinitis/NPC=3/10/6. Scale bar in upper and middle panels: 50 μm. Scale bar in lower panel: 100 μm. Quantification of FSP1^+^ (brown), CD163^+^ (brown), CD31^+^ (red), and NG2^+^ (green) and coverage rate of NG2^+^ pericytes (*n =* 8 random fields per group). (**G**) qPCR quantification of *FGF2*, *CD163*, *CD31*, *NG2*, and *FSP1* mRNA in freshly collected tissues. Sample number: Rhinitis/NPC=5/6. (**H**) Correlation of *FGF2* and *CD163* expression of human NPCs and their control healthy tissues. Sample number: Control/NPC=10/31. *P < 0.05, **P < 0.01, ***P < 0.001 by unpaired 2-tailed Student’s *t* test (**B**, **D**, **E**, **G**, and **H**) or 1-way ANOVA with Tukey’s multiple-comparison analysis (**C**, **D**, and **F**). Data are presented as mean ± SD.

In the TME, various signaling molecules orchestrate cancer metastasis via autocrine, paracrine, and endocrine mechanisms (14). To investigate the molecular mechanism of NPC metastasis, we applied a gene expression profiling analysis associated with metastasis-related growth factors to screen NPC and various other cancer types. Twenty-three metastasis-related growth factors were selected for the screening. Tissue RNA expression data sets of major cancer types including colon adenocarcinoma (COAD), breast invasive carcinoma (BRCA), lung adenocarcinoma (LUAD), ovarian cancer (OV), stomach adenocarcinoma (STAD), skin cutaneous melanoma (SKCM), pancreatic adenocarcinoma (PAAD), liver hepatocellular carcinoma (LIHC), and kidney renal clear cell carcinoma (KIRC) and their corresponding controls were downloaded from The Cancer Genome Atlas (TCGA). Tissue RNA expression data sets of NPC and its corresponding control with accession no. GSE12452 were downloaded from the Gene Expression Omnibus (GEO). We compared a panel of selected genes among these expression profiles in each tumor type with its respective adjacent control tissues. Interestingly, *VEGFB* in NPC showed similar expression levels among various cancers. *VEGFC* and *TNFB* were expressed at low levels in NPC but high levels in most of the non-NPC cancer types. Surprisingly, *FGF2*, a potent mitogenic factor of the FGF family, was exclusively highly expressed in NPC (Figure 1A). TCGA analysis further confirmed the lower *FGF2* expression in non-NPC cancer tissues (Figure 1B and Supplemental Figure 1, A and B; supplemental material available online with this article; https://doi.org/10.1172/jci.insight.157874DS1). Next, we applied the correlation study of *FGF2* expression in various clinical stages of NPCs compared with normal nasopharyngeal tissue (NNT) using the GSE12452 data set containing 31 NPC tissue specimens and 10 healthy NNT specimens. It demonstrated the higher expression of *FGF2* in all stages of NPCs (Figure 1C). No expression difference among various NPC stages was observed (Figure 1C).

To further identify the cell type origin of FGF-2 in NPC tissues, we collected 3 NNTs, 10 rhinitis tissues, and 6 NPC tissues from patients receiving a nasopharyngoscopy test. The demographic information of these patients was shown (Supplemental Table 1). Histological analysis showed significantly high FGF-2 expression in NPC tissues compared with non-NPC tissues. Quantification analysis under the supervision of an experienced pathologist showed over a 7-fold increase of FGF-2^+^ signals in NPCs relative to NNTs or to rhinitis tissues (Figure 1D). Moreover, the major cellular component expressing FGF-2 in the NPC microenvironment was epithelial cells, indicating that FGF-2 originated from NPC cancer cells (Figure 1D). To further validate the source of FGF-2 production in the TME, we applied a negative selection strategy to isolate NPC cancer cells without knowing cancer cell surface marker expression. Cancer cells were isolated from 3 fresh NPC tissues using a magnetic-activated cell sorting (MACS) kit. As expected, cancer cells showed significantly high FGF-2 expressions (Figure 1E). These results demonstrate the distinct FGF-2 expression in NPC cancer cells.

To investigate potential structural changes induced by FGF-2 signaling, various cellular components were analyzed by staining with fibroblast-specific protein 1^+^ (FSP1^+^), CD163^+^ signals, CD31^+^, and neuron-glial antigen 2^+^ (NG2^+^) signals. Interestingly, fibroblasts were not abundant cellular components in NPC tumor tissues (Figure 1F). In contrast, macrophage infiltration into NPC tumor tissues was highly increased. Quantification analysis showed that over a 4-fold increase of CD163^+^ signals was found in NPCs relative to NNTs (Figure 1F). A higher number of vessels was observed in NPCs, and the pericyte coverage of these vessels is similar to that in NNTs (Figure 1F). These data demonstrate a significant increase of TAM infiltration in NPC tissues. To further validate that these findings are specific to NPC tissue, we collected 5 rhinitis fresh tissues and 6 NPC fresh tissues. Indeed, *FGF2* and *CD163* were significantly expressed in NPC tissues compared with rhinitis tissues. There was no change of *FSP1* expression between these 2 groups (Figure 1G). In GSE12452, analysis revealed an impeccable correlation between *FGF2* and *CD163* expression, suggesting an FGF-2–induced inflammation (Figure 1H). These data show that NPC-derived FGF-2 correlates with TAMs infiltration in the TME.

### FGF-2 promotes NPC metastasis.

**Figure 2:**
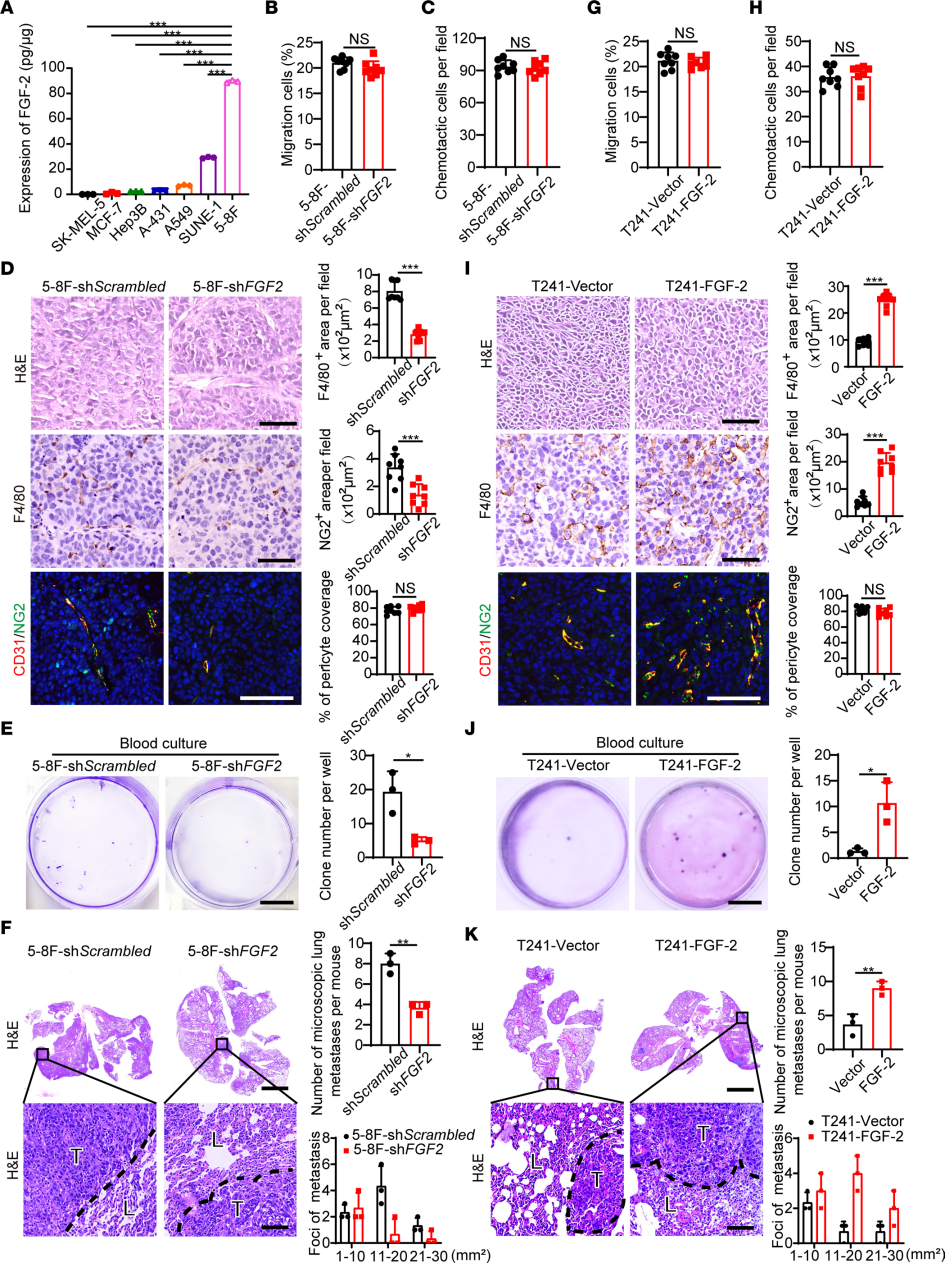
FGF-2 promotes TAM infiltration and tumor metastasis in mice. (**A**) FGF-2 protein expression levels in various human tumor cell lines, including SK-MEL-5 melanoma, MCF-7 breast cancer, Hep3B hepatocellular carcinoma, A-431 squamous cell carcinoma, A549 lung cancer, SUNE-1 NPC, and 5-8F NPC (*n =* 3 samples per group). (**B**, **C**, **G**, and **H**) Migration and chemotactic ability of scrambled and *FGF2* shRNA–transfected NPC cancer cells (**B** and **C**) and of vector and FGF-2 overexpressing T241 tumor cells (**G** and **H**). (**D** and **I**) Xenograft tumor tissues were stained with H&E, an anti-F4/80 antibody (brown), an anti-CD31 antibody, and an anti-NG2 antibody (*n =* 8 mice per group). Scale bar in upper panel: 50 μm. Scale bar in middle panel: 50 μm. Scale bar in lower panel: 100 μm. Quantification of F4/80^+^ signals, NG2^+^ signals, and coverage rate of NG2^+^ pericytes (*n =* 8 random fields per group). (**E** and **J**) Micrographs of representative cell culture dishes after incubation with blood samples from 5-8F sh*Scrambled* or 5-8F sh*FGF2* tumor–bearing mice (**E**) and from vector or FGF-2–overexpressing tumor–bearing mice (**J**). Blue signal indicates the crystal violet–positive tumor colonies. Scale bar: 1 cm (*n =* 3 samples randomly chosen from 8 mice per group). (**F** and **K**) H&E staining in the lung from 5-8F sh*Scrambled* or 5-8F sh*FGF2* tumor–bearing mice (**F**) and from vector or FGF-2–overexpressing tumor–bearing mice (**K**). Scale bar in upper panel: 3 mm. Scale bar in lower panel: 100 μm. Quantification of total microscopic lung metastases and various sizes of metastases (*n =* 3 samples randomly chosen from 8 mice per group). *P < 0.05, **P < 0.01, ***P < 0.001 by unpaired 2-tailed Student’s *t* test (**B**–**K**) or 1-way ANOVA with Tukey’s multiple-comparison analysis (**A**). Data are presented as mean ± SD.

We then tested FGF-2 protein levels in various human tumor cell lines. Compared with melanoma, breast cancer, hepatocellular carcinoma, squamous cell carcinoma, and lung cancer cell lines, NPC cell lines SUNE-1 and 5-8F showed dramatically high levels of FGF-2 (Figure 2A), validating the results from clinical NPC samples and the database analysis. To investigate the role of FGF-2 in promoting tumor growth, recruiting TAMs, and metastasis in NPC tumors, we next chose natural FGF-2 high-expressing human NPC 5-8F cells and performed *FGF2*-specific short hairpin RNA–knockdown (shRNA-knockdown) experiments. As expected, stable transfection of *FGF2*-specific shRNA effectively suppressed FGF-2 production (Supplemental Figure 2, A and B). Knockdown of *FGF2* reduced tumor cell growth rates without affecting migration ability compared with the scrambled shRNA–transfected control tumor cells (Supplemental Figure 2, C and D, and Figure 2, B and C). Interestingly, *FGF2* shRNA–transfected 5-8F tumors in mice lacked TAM infiltration and reduced vascular-associated pericytes compared with the control tumors (Figure 2D). In addition, *FGF2* knockdown markedly suppressed circulating tumor cells (CTCs), tumor clones in blood culture, and pulmonary metastasis (Figure 2, E and F, and Supplemental Figure 2, E and F). Pulmonary metastases were validated and quantified using gross examination, ex vivo visualization, and H&E analysis (Figure 2F and Supplemental Figure 2F). To further validate our results, we performed a gain-of-function model in which mouse T241 tumors were genetically propagated to stably express human FGF-2. Transfected cells expressed high levels of FGF-2 in both mRNA and protein levels (Supplemental Figure 2, G and H). FGF-2 overexpression facilitated tumor cell growth without changing migration ability (Supplemental Figure 2, I and J, and Figure 2, G and H). Of note, FGF-2–expressing tumors contained a high density of F4/80^+^ TAMs, microvessels, and vascular-associated pericytes (Figure 2I). Interestingly, FGF-2–expressing tumor–bearing mice showed markedly higher CTCs, tumor clones in blood culture, and pulmonary metastasis, compared with that in vector-transfected controls (Figure 2, J and K, and Supplemental Figure 2, K and L). Using gain- and loss-of-function models, we provide compelling evidence that FGF-2 contributes to promoting angiogenesis, TAMs infiltration, and tumor metastasis.

### FGF-2 drives macrophage migration via pericyte-secreted factors.

**Figure 3:**
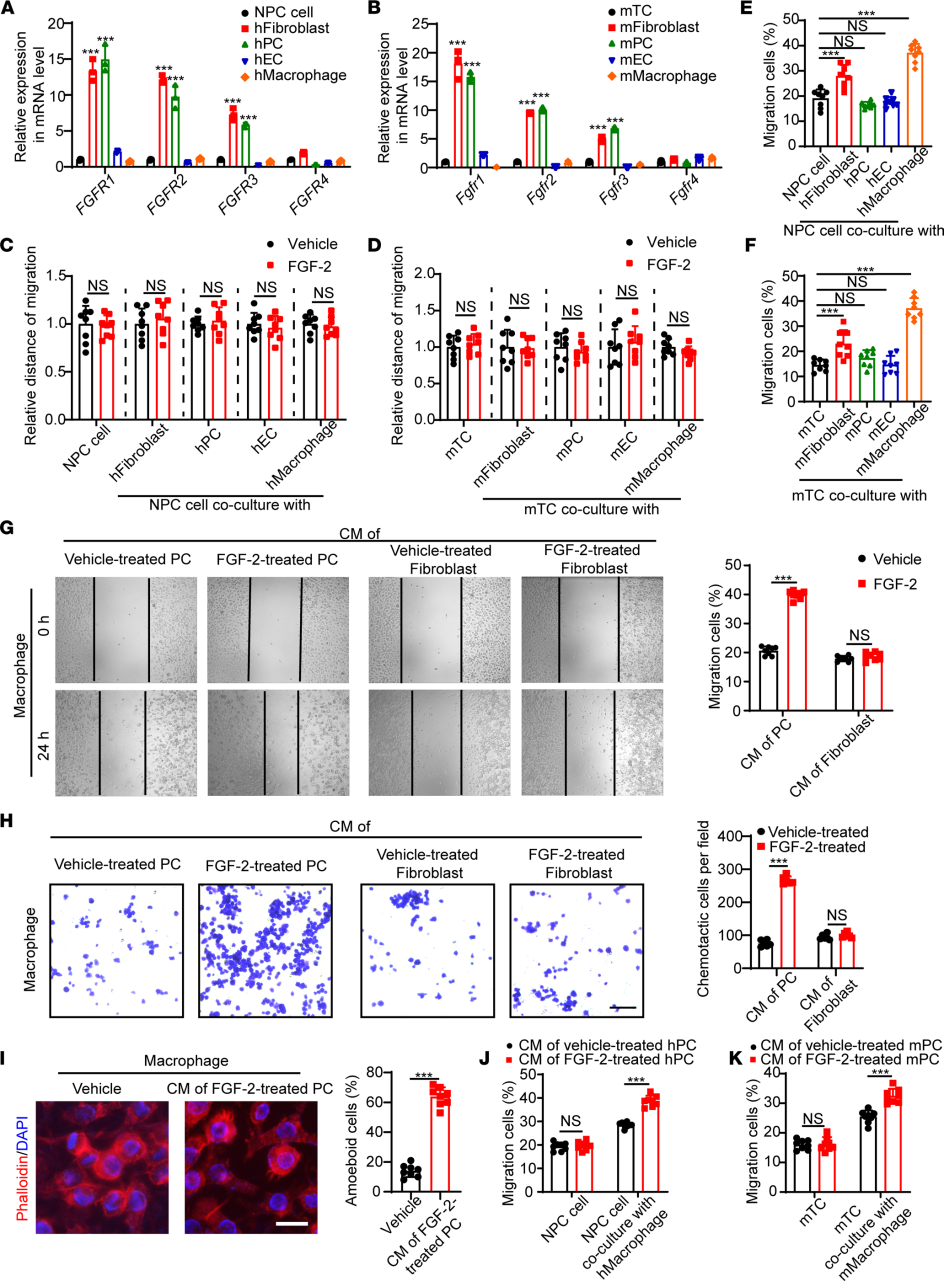
Pericyte-dependent mechanism of FGF-2–induced macrophage activation. (**A** and **B**) qPCR quantification of human and mouse *FGFR1*, *FGFR2*, *FGFR3*, and *FGFR4* mRNA levels in various cell types, including 5-8F NPC cell line, hTERT-immortalized dermal fibroblasts, isolated primary pericytes, HUVEC endothelial cells, THP-1 monocyte/macrophage cell line, mouse T241 cell line, mouse MS5 stromal fibroblasts, mouse lung isolated primary pericytes, mouse liver isolated primary endothelial cells, and murine RAW 264.7 monocyte/macrophage cell line. (**C** and **D**) Human and mouse tumor cell migration of tumor cells cocultured with various cell types in the presence or absence of FGF-2. Vehicle- or FGF-2–treated tumor cells serve as controls (*n =* 8 samples per group). (**E** and **F**) Human and mouse tumor cell migration of tumor cells cocultured with or without various cell types (*n =* 8 samples per group). (**G** and **H**) Conditioned medium of pericytes or fibroblasts in the presence or absence of FGF-2 was collected. Mouse macrophage migration (*n =* 8 samples per group) and chemotactic ability (*n =* 6 samples per group) of macrophages treated with various conditioned medium are shown. (**I**) Morphological changes of macrophage administrated with vehicle or the conditioned medium of FGF-2–treated pericytes. Quantification of macrophage structural changes (*n =* 8 random fields per group). (**J** and **K**) Human and mouse tumor cell migration of tumor cells cocultured with macrophages, which activated with FGF-2–treated pericyte conditioned medium. Tumor cells receiving the FGF-2–treated pericyte conditioned medium serve as controls (*n =* 8 samples per group). ***P < 0.001 by unpaired 2-tailed Student’s *t* test (**C**, **D**, and **G**–**K**) or 1-way ANOVA with Tukey’s multiple-comparison analysis (**A**, **B**, **E**, and **F**). Data are presented as mean ± SD.

We next analyzed various cell types to identify FGFR expression. In both human and mouse cells, *FGFR1–3* were highly expressed in both fibroblasts and pericytes (Figure 3, A and B). Of note, FGF-2 stimulated the proliferation of pericytes and fibroblasts from both human and mouse origins (Supplemental Figure 3A). These results are in agreement with published literature (21). Only marginal expression levels were found in tumor cells, endothelial cells, and macrophages (Figure 3, A and B). These findings show that fibroblasts and pericytes in the tumor stroma — but not tumor cells, per se — express FGFRs. This is consistent with the fact that FGF-2 knockdown in NPC cells did not affect their migratory capacity (Figure 2, B and C). Since fibroblasts and pericytes distinctively express receptors of FGF-2 in the TME, we hypothesized that FGF-2–promoted tumor metastasis requires assistance from fibroblasts or pericytes. To test that hypothesis, we treated various cells with FGF-2 and cocultured them with tumor cells. Surprisingly, in both human NPC cells and mouse tumor cells, coculture with FGF-2–stimulated fibroblasts or pericytes did not enhance the tumor migration rate in vitro (Figure 3, C and D), suggesting a more complex interaction among cell components. To investigate fibroblasts and pericytes highly expressing FGFR in the tumor metastasis process, we next examined the contribution of various cell types to tumor cell migration using a coculture system. We found that tumor cells cocultured with macrophages increased their migration by 2 fold, suggesting a macrophage-tumor cell interaction mechanism of metastasis (Figure 3, E and F). Given the fact that macrophages did not express FGFRs (Figure 3, A and B) and that FGF-2 stimulation did not alter macrophage migration (Supplemental Figure 3, B and C), we hypothesized that FGF-2 indirectly activates macrophages via fibroblasts or pericytes for tumor cell migration. We collected the conditioned medium of FGF-2–treated fibroblasts or pericytes, and we stimulated macrophages with these media. Surprisingly, significant morphological changes and a markedly increased migration rate of macrophages were observed only in the pericyte-conditioned medium group (Figure 3, G–I), suggesting that macrophages can be activated by pericyte-derived, but not fibroblast-derived, factors. Furthermore, precultured with this macrophage-activating medium, macrophages strongly drove tumor migration (Figure 3, J and K). These results suggest an FGF-2/pericyte/macrophage/tumor cell axis in tumor migration.

### FGF-2 induces pericyte-derived CXCL14 via FGFR1/AHR signaling.

**Figure 4:**
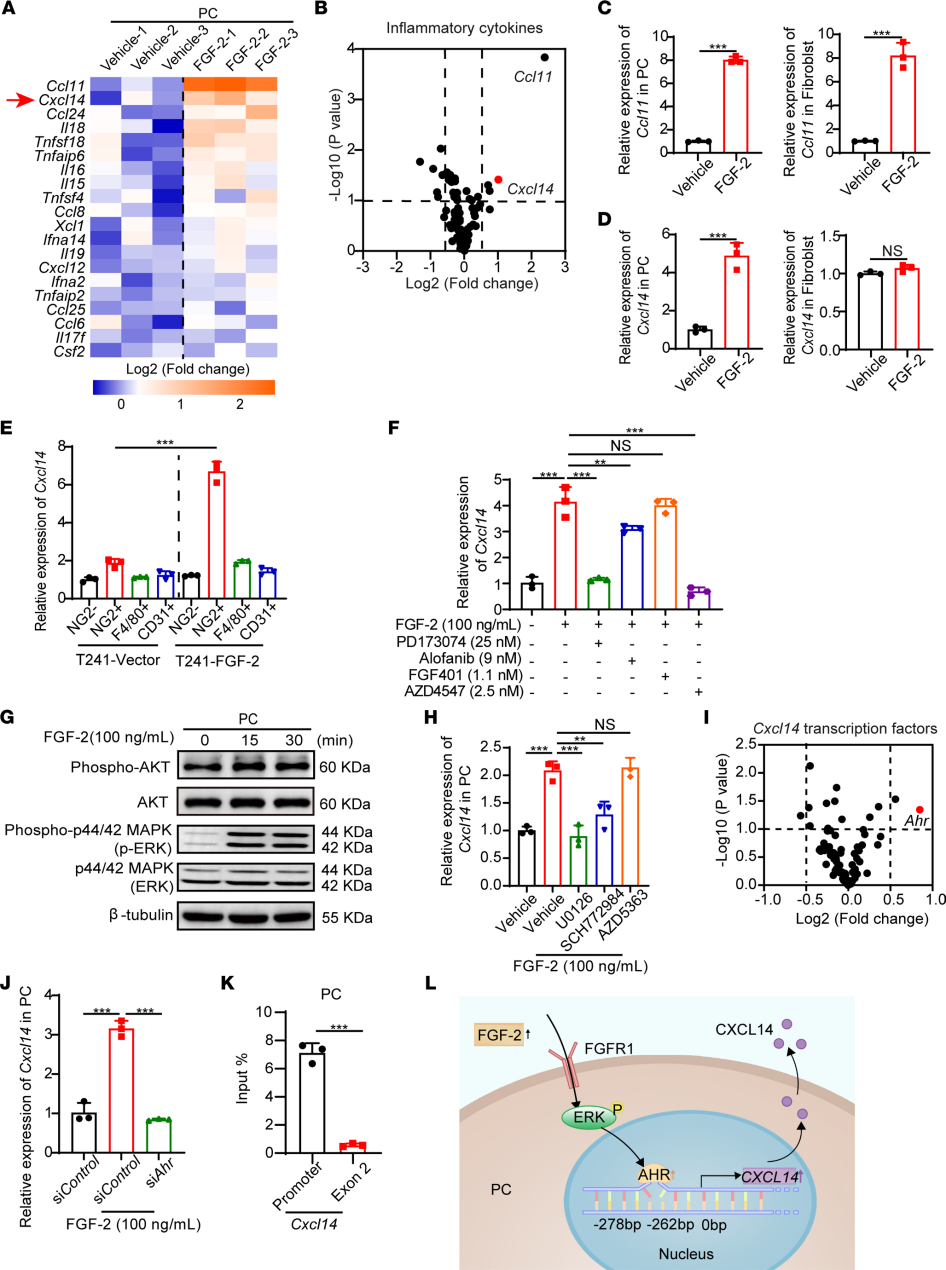
FGF-2 induces CXCL14 expression in pericytes via FGFR1/ERK/AHR signaling. (**A**) Heatmap of selected genes by inflammatory cytokine/chemokine profiling of vehicle- and FGF-2–treated primary mouse pericytes (*n =* 3 samples per group). Arrow points to upregulated *Cxcl14* gene. (**B**) Volcano plot of inflammatory gene profiling of vehicle- and FGF-2–stimulated pericytes (*n =* 3 samples per group). (**C** and **D**) Expression levels of *Ccl11* and *Cxcl14* in vehicle- and FGF-2–stimulated isolated primary pericytes and MS5 fibroblasts (*n =* 3 samples per group). (**E**) qPCR quantification of *Cxcl14* mRNA levels in F4/80^+^ TAMs, NG2^+^ pericytes, CD31^+^ endothelial cells, and NG2^–^ population isolated from T241-vector and T241–FGF-2 tumors (*n =* 3 samples per group). (**F**) qPCR quantification of *Cxcl14* mRNA levels in vehicle- and FGF-2–stimulated pericytes in the presence or absence of FGFR1, FGFR2, and FGFR3 specific inhibitors, and pan-FGFR inhibitor (*n =* 3 samples per group). (**G**) After 0, 15, 30 minutes of stimulation, FGF-2 induced phosphorylation of AKT and ERK in pericytes. β-Tubulin marks the loading level in each lane. These experiments were repeated twice. (**H**) qPCR quantification of *Cxcl14* mRNA levels in vehicle- and FGF-2–stimulated pericytes in the presence or absence of MEK1/2, ERK1/2, and AKT specific inhibitors (*n =* 3 samples per group). (**I**) Volcano plot of predicted transcription factors which bind to *Cxcl14* promoter in genome-wide expression profiling of vehicle- and FGF-2–stimulated pericytes (*n =* 3 samples per group). (**J**) qPCR quantification of *Cxcl14* mRNA levels in vehicle- and FGF-2–stimulated pericytes in the presence or absence of Control or Ahr-specific siRNA (*n =* 3 samples per group). (**K**) ChIP assay of AHR binding to the *Cxcl14* gene promoter. Nonimmune IgG and *Cxcl14* exon 2 regions served as controls (*n =* 3 samples per group). (**L**) Mechanistic diagram of the FGF-2/FGFR1/ERK/AHR/CXCL14 signaling pathway. **P < 0.01, ***P < 0.001 by unpaired 2-tailed Student’s *t* test (**C**–**E** and **K**) or 1-way ANOVA with Tukey’s multiple-comparison analysis (**F**, **H**, and **J**). Data are presented as mean ± SD.

To identify possible factors that mediate FGF-2–induced macrophage activation, we performed an inflammatory cytokine/chemokine profiling based on genome-wide expression microarray analysis in freshly isolated pericytes in vector- and FGF-2–overexpressing tumors. Interestingly, *Ccl11* and *Cxcl14* ranked as the top 2 upregulated genes (Figure 4, A and B). The FGF-2–induced *Ccl11* and *Cxcl14* expression was further validated (Figure 4, C and D). Of note, *Ccl11* was upregulated in both FGF-2–stimulated pericytes and fibroblasts, whereas *Cxcl14* was upregulated only in FGF-2–stimulated pericytes (Figure 4, C and D). In addition, the major receptor for CCL11, CCR3 (25), was barely expressed in macrophages but was highly expressed in basophils and eosinophils (Supplemental Figure 4A). These results suggest that CXCL14, rather than CCL11, may mediate the pericyte-specific activation of macrophages. To further validate the pericytes as the major sources of CXCL14 production in an in vivo model, we isolated host cells — including pericytes, TAMs, and endothelial cells — from the TME using NG2, F4/80, and CD31 markers (Supplemental Figure 4, B–D). We confirmed that the NG2^+^ cell population was the critical cell type to produce *Cxcl14* in FGF-2–high tumors (Figure 4E). In contrast, as a downstream executor of the pericyte-macrophage axis, F4/80^+^ macrophages did not contribute to FGF-2–induced *Cxcl14* expression (Figure 4E). Similarly, the NG2^–^ cell population, including tumor cells, produced negligible levels of *Cxcl14* in both FGF-2^+^ and FGF-2^–^ tumors (Figure 4E). These findings demonstrate that pericytes are the primary source of CXCL14 in the FGF-2–expressing TME.

Next, we investigated the engaged receptors and signaling pathway by which FGF-2 induces CXCL14 expression in tumoral pericytes. FGF-2–stimulated pericytes were treated with FGFR inhibitors, including FGFR1 selective inhibitor PD173074, FGFR2 selective inhibitor alofanib, FGFR4 selective inhibitor FGF401, and FGFR paninhibitor AZD4547. Interestingly, FGFR1 inhibitor completely blocked the *Cxcl14* production, while paninhibition of FGFRs did not produce a further inhibitory effect (Figure 4F), suggesting that FGFR1 is responsible for FGF-2–induced *Cxcl14* expression. Similar to published studies, FGF-2 strongly drove ERK phosphorylation but not AKT phosphorylation (Figure 4G). Inhibition of MEK or ERK1/2 by selective inhibitors U0126 or SCH772984 blocked FGF-2–induced *Cxcl14* expression in pericytes, while AKT inhibitor AZD5363 did not affect *Cxcl14* expression (Figure 4H). These results suggest that ERK is involved in FGF2-induced CXCL14 expression.

We further analyzed the potential transcription factors regulating *Cxcl14* using a prediction tool PROMO (26). Genome-wide microarray analysis of FGF-2–stimulated pericytes revealed that, in all the potential regulators of *Cxcl14*, *aryl hydrocarbon receptor* (*Ahr*) was the most upregulated transcription factor (Figure 4I). Indeed, FGF-2–stimulated pericytes, rather than fibroblasts, expressed a high level of *Ahr*, supporting that AHR confers pericyte-specific *Cxcl14* expression (Supplemental Figure 4E). The increased expression was further validated by quantitative PCR (qPCR) (Figure 4J). Knockdown of *Ahr* using siRNA significantly impaired FGF-2–induced *Cxcl14* expression (Figure 4J and Supplemental Figure 4F). To provide experimental evidence for validating how AHR physically interacts with the *Cxcl14* promoter, we analyzed the mouse *Cxcl14* promoter region and discovered a canonical AHR-binding site at –278 bp. ChIP assay using the *Cxcl14* promoter fragment containing the binding site and exon fragment demonstrated that AHR binds to the *Cxcl14* promoter (Figure 4K). These findings suggest an FGF-2/FGFR1/AHR/CXCL14 axis in pericytes (Figure 4L).

### CXCL14 promotes TAM infiltration and polarization.

**Figure 5:**
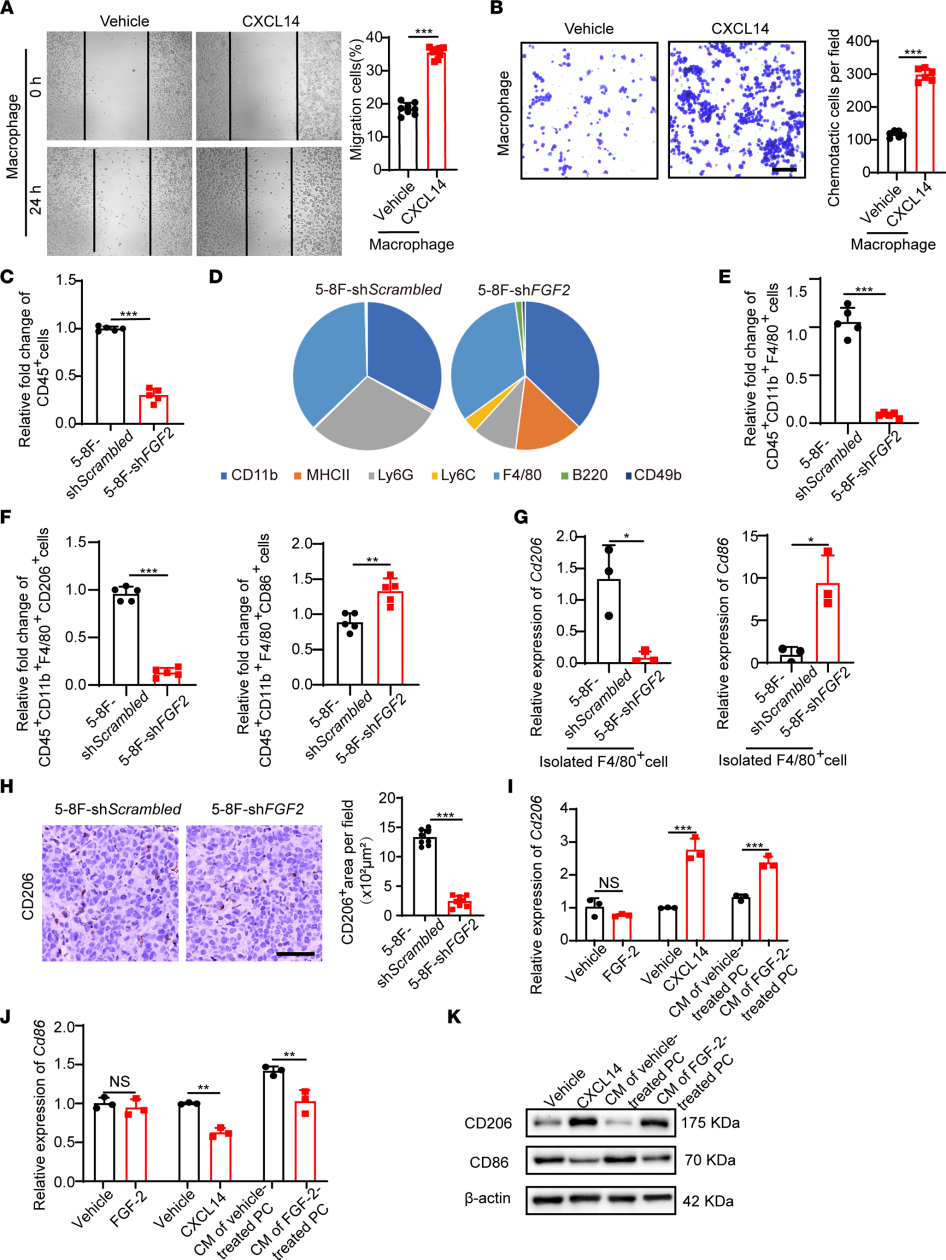
CXCL14 recruits, activates, and polarizes TAMs. (**A** and **B**) Mouse macrophage migration (*n =* 8 samples per group) and chemotactic ability (*n =* 6 samples per group) of macrophage treated with or without CXCL14. (**C**) Quantification of CD45^+^ cells in xenograft sh*Scrambled*- and sh*FGF2*-transfected NPC tumors (*n =* 5 samples per group). (**D**) Pie charts of percentage of various inflammatory cells in xenograft sh*Scrambled*- and sh*FGF2*-transfected NPC tumors (*n =* 5 samples per group). CD45^+^CD11b^+^F4/80^+^ macrophage population, CD45^+^MHCII^+^CD11b^+^CD11c^+^ DC population, CD45^+^CD11b^+^Ly6G^hi^Ly6C^int^ granulocytic subsets of myeloid-derived suppressor cell population, CD45^+^CD11b^+^Ly6G^–^Ly6C^+^ monocytic subsets of myeloid-derived suppressor cell population, CD45^+^B220^+^ B cell population, and CD45^+^CD11b^–^CD49b^+^ NK cell population were analyzed. (**E** and **F**) Quantification of CD45^+^CD11b^+^F4/80^+^ TAM population, CD45^+^CD11b^+^ F4/80^+^CD206^+^ M2-like TAM population, and CD45^+^CD11b^+^F4/80^+^CD86^+^ M1-like TAM population (*n =* 5 sample per group). (**G**) qPCR quantification of *CD206* and *CD86* mRNA levels in F4/80^+^ TAMs isolated from xenograft sh*Scrambled*- and sh*FGF2*-transfected NPC tumors (*n =* 3 samples per group). (**H**) Tumor tissues were stained with an anti-CD206 antibody (brown). Scale bar: 50 μm. Quantification of CD206^+^ signals (*n =* 8 random fields per group). (**I** and **J**) qPCR quantification of *CD206* and *CD86* mRNA levels in macrophages that were activated with FGF-2–treated pericyte conditioned medium or CXCL14. Vehicle- and FGF-2–stimulated macrophages serve as controls (*n =* 3 samples per group). (**K**) CXCL14- or FGF-2–treated pericyte conditioned medium–induced CD206 upregulation and CD86 downregulation in macrophages. β-Actin marks the loading level in each lane. These experiments were repeated twice. *P < 0.05, **P < 0.01, ***P < 0.001 by unpaired 2-tailed Student’s *t* test (**A**–**C** and **E**–**J**). Data are presented as mean ± SD.

To investigate the functional impact of CXCL14 on macrophages, we performed in vitro experiments on macrophage migration via wound healing assay and chemotaxis assay. As expected, CXCL14 significantly recruited macrophages and promoted migration (Figure 5, A and B). It is known that, in the TME, TAMs with M2 phenotype are associated with tumor growth and invasion (27). Although FGF-2 is correlated with TAM infiltration in NPCs (Figure 1, F–H), the phenotypic characteristics of TAMs have not been identified. We extracted an equal amount of scrambled control- and *FGF2* shRNA–transfected 5-8F tumor tissues and performed FACS analysis of various immune cells (Supplemental Figure 5A). The number of CD45^+^ cells was significantly decreased by two-thirds in sh*FGF2* NPC tumors (Figure 5C), validating the inflammatory effect of FGF-2 in the TME. Interestingly, the proportion of F4/80^+^ cells in the composition of the various types of immune cells did not change (Figure 5D), suggesting that the inflammatory effect of FGF-2 is not limited in macrophages. Notably, MHCII^+^ cells occupied a greater proportion in the *FGF2* shRNA–transfected 5-8F TME compared with that in the control group (Figure 5D), suggesting that FGF-2 affects DCs infiltration (Supplemental Figure 5B). Further FACS analysis showed a dramatic reduction of total TAMs (Figure 5E). Surprisingly, CD206^+^ M2-like TAMs, but not CD86^+^ M1-like TAMs, were reduced in the FGF-2 knockdown group (Figure 5F), supporting the role of FGF-2 on TAMs polarization toward M2 phenotype. To validate these FACS results, we isolated F4/80^+^ cells using MACS and then detected *CD206* and *CD86* RNA expression. Knockdown of FGF-2 in the TME dramatically reduced *CD206* expression in TAMs (Figure 5G). IHC staining showed a similar CD206 reduction in NPC xenograft tumor tissues (Figure 5H). These findings demonstrate that FGF-2 promotes TAMs recruitment and polarization toward the M2 phenotype.

We next analyzed the role of CXCL14 on macrophage polarization. Indeed, direct stimulation of macrophages with CXCL14 significantly increased *CD206* expression and decreased *CD86* expression, while FGF-2 did not alter the expression of these markers (Figure 5, I and J). In contrast, using the conditioned medium of FGF-2–stimulated pericytes, we could reproduce CXCL14-induced macrophage polarization (Figure 5, I and J). We further verified the polarization effect of CXCL14 at the protein level (Figure 5K). These results indicate that pericyte-derived CXCL14 is the mediator of FGF-2–induced macrophage recruitment and polarization.

### Selective depletion of pericytes prevents CXCL14 expression and TAM infiltration.

**Figure 6:**
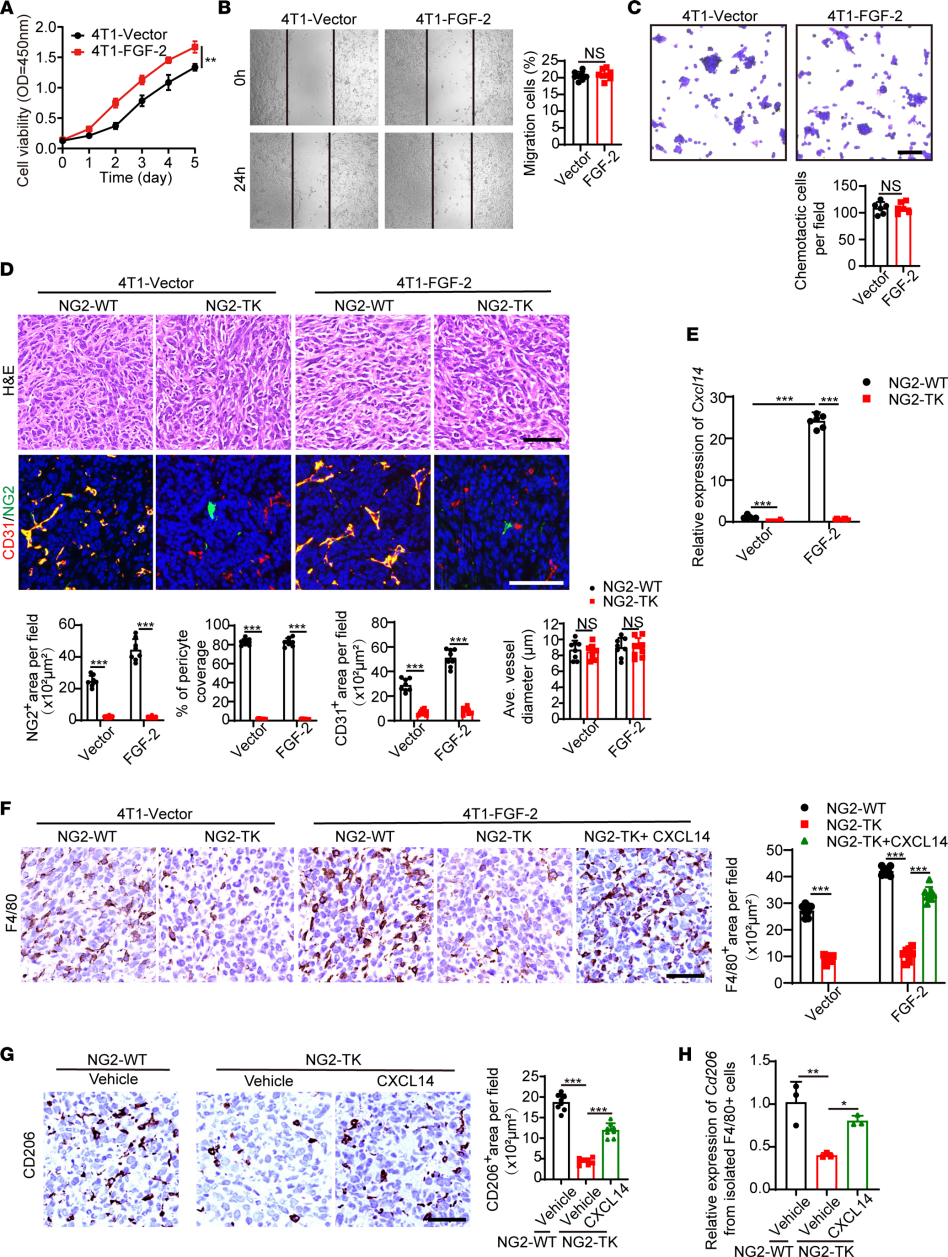
Genetic depletion of pericytes ablates CXCL14 and TAM infiltration in the TME. (**A**) Growth rates of 4T1-vector and 4T1–FGF-2–overexpressing tumor cells in vitro. (**B** and **C**) Cell migration (*n =* 8 samples per group) and chemotactic ability (*n =* 6 samples per group) of 4T1-vector and 4T1–FGF-2–overexpressing tumor cells. (**D**) Tumor-bearing WT and NG2-TK mice were administrated with ganciclovir when the tumor reached 0.5 cm^3^. H&E staining and immunofluorescence localization of CD31 (red), NG2 (green), and DAPI (blue) signals in 4T1-vector and 4T1-FGF-2–overexpressing tumor–bearing WT and NG2-TK mice (*n =* 6 mice per group). Scale bar in upper panel: 50 μm. Scale bar in lower panel: 100 μm. Quantification of CD31^+^ signals, NG2^+^ signals, pericyte coverage, and average vessel diameters (*n =* 8 random fields per group). (**E**) qPCR quantification of *Cxcl14* mRNA levels of 4T1-vector and 4T1–FGF-2–overexpressing tumor tissues from WT and NG2-TK mice (*n =* 6 mice per group). (**F**) F4/80 (brown) IHC in vector and FGF-2 tumor with or without NG2^+^ pericyte depletion and in CXCL14-administrated, NG2^+^ pericyte–depleted FGF-2 tumor (*n =* 6 mice per group). Scale bar: 50 μm. Quantification of F4/80^+^ signals (*n =* 8 random fields per group) (**G**) CD206 (brown) IHC in FGF-2 tumor with or without NG2^+^ pericyte depletion or CXCL14 administration (*n =* 6 mice per group). Scale bar: 50 μm. Quantification of CD206^+^ signals (*n =* 8 random fields per group) (**H**) qPCR quantification of *Cd206* mRNA levels in F4/80^+^ TAMs from various tumor groups (*n =* 3 samples per group). *P < 0.05, **P < 0.01, ***P < 0.001 by unpaired 2-tailed Student’s *t* test (**A**–**D**) or 1-way ANOVA with Tukey’s multiple-comparison analysis (**E**–**H**). Data are presented as mean ± SD.

To study the inflammatory impact of pericytes on TAMs in vivo, we applied an NG2–thymidine kinase (NG2-TK) mouse model in which chondroitin sulfate proteoglycan 4 (*Cspg4*, *Ng2*) gene promoter controls the expression of herpes simplex 1 virus TK in BALB/c mice. This strain allows ganciclovir-inducible ablation of NG2^+^ pericytes (28). Due to the BALB/c background of this strain, we constructed another tumor cell model that is compatible with BALB/c background, mouse breast cancer 4T1, and stably transfected with human FGF-2 or empty vectors. This tumor cell line pair recapitulate the results from human 5-8F and mouse T241 pairs in vitro (Figure 6, A–C). Interestingly, similar to previous results, 4T1–FGF-2 tumors in WT mice show increased vasculatures with pericyte coverage compared with the 4T1-vector control group (Figure 6D). In NG2-TK mice, after ganciclovir treatment, tumoral pericytes were completely ablated (Figure 6D). Of note, vasculature density was significantly reduced without altering the vessel diameter (Figure 6D), probably due to the high interstitial pressure in the TME. To detect the CXCL14 production levels in tumors, we collected the total RNA of tumor tissue and performed qPCR analysis. *Cxcl14* was significantly expressed in FGF-2–expressing tumors and was down to an undetectable level after pericytes ablation (Figure 6E). These results provide compelling evidence that FGF-2 specifically promotes CXCL14 expression in NG2^+^ pericytes and that NG2^+^ pericytes are the critical source of CXCL14 in vivo.

We further investigated the role of pericyte and the role of CXCL14 in TAM infiltration. As expected, in vector tumors, TAMs were observed, and removal of pericytes further reduced them to trace amounts (Figure 6F). Compared with vector tumors, overexpression of FGF-2 significantly promoted TAMs infiltration, while depletion of pericytes completely blocked TAMs (Figure 6F). These results confirmed that pericytes in the FGF-2 TME are critical for TAM infiltration. To further investigate the role of CXCL14 on TAM infiltration in vivo, we injected CXCL14 protein intratumorally. Interestingly, CXCL14 administration significantly increased TAMs in pericyte-depleted FGF-2 tumors (Figure 6F). These CXCL14-recruited TAMs were further isolated and identified as CD206^+^ M2 phenotype (Figure 6, G and H). Given that pericytes have been shown to be the sole CXCL14 source in the FGF-2 TME (Figure 4E and Figure 6E), these results suggest that pericyte-derived CXCL14 promotes the recruitment and M2-polarization of TAMs in vivo.

Next, we explored metastatic activities in this pericyte depletion model. Interestingly, although ganciclovir ablated *Cxcl14* expression and TAM infiltration, tumor metastasis was increased (Supplemental Figure 6, A–C). One would reasonably speculate that decrease of TAM may reduce tumor metastasis. However, in addition to immune regulation, pericytes also play an important role in vascular coating. Various studies from other groups and our group have shown that pericyte ablation increases vascular leakage and, hence, tumor metastasis (29). These results suggest that a simple deletion of pericytes to treat tumor metastasis is not ideal. Instead, targeting the later steps of the FGF-2/pericyte/CXCL14/TAMs axis might be a better approach.

### TAM-dependent metastasis of high FGF-2 tumors.

**Figure 7:**
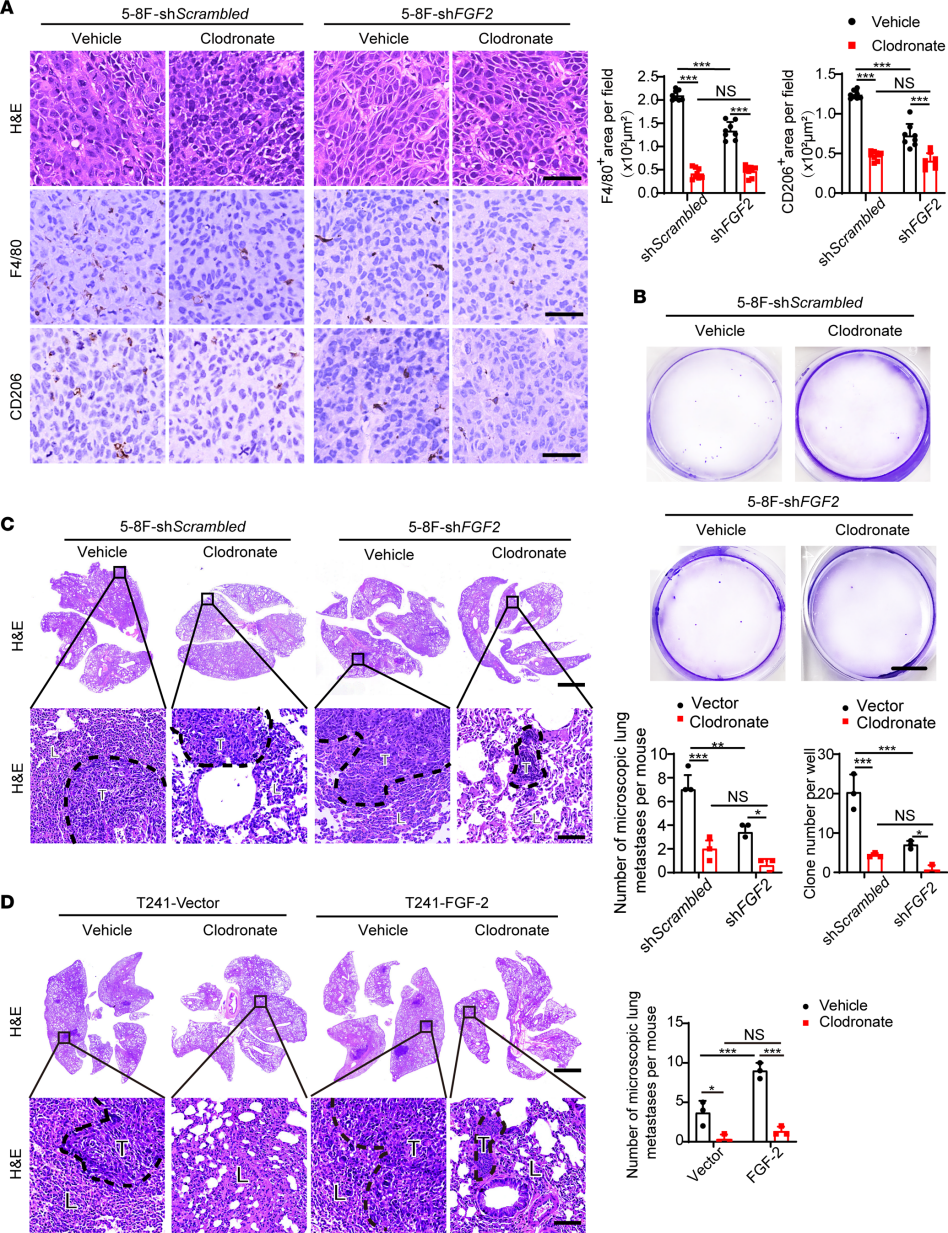
Pharmacological TAM depletion diminishes FGF-2–induced NPC metastasis. (**A**) Micrographs of H&E and IHC staining with F4/80 (brown) or CD206 (brown) in 5-8F sh*Scrambled* or 5-8F sh*FGF2* tumors implanted in clodronate-treated and nontreated mice. Scale bar: 50 μm. Quantification of F4/80^+^ and CD206^+^ signals (*n =* 8 random fields per group). (**B**) Micrographs of representative cell culture dishes after incubation with blood samples from 5-8F sh*Scrambled* or 5-8F sh*FGF2* tumor–bearing mice receiving vehicle or clodronate liposomes. Blue signal indicates the crystal violet-positive tumor colonies. Scale bar: 1 cm. (**C**) H&E staining in the lung from 5-8F sh*Scrambled* or 5-8F sh*FGF2* tumor–bearing mice. Scale bar in upper panel: 3 mm. Scale bar in lower panel: 100 μm. Quantification of total microscopic lung metastases and various sizes of metastases (*n =* 3 samples randomly chosen from 6 mice per group). (**D**) H&E staining in the lung from vector or FGF-2–overexpressing tumor–bearing mice. Scale bar in upper panel: 3 mm. Scale bar in lower panel: 100 μm. Quantification of total microscopic lung metastases and various sizes of metastases (*n =* 3 samples randomly chosen from 6 mice per group). **P < 0.01, ***P < 0.001 by 1-way ANOVA with Tukey’s multiple-comparison analysis (**A**–**D**). Data are presented as mean ± SD.

We next investigated the impact of TAMs in promoting metastasis using a pharmacological approach. To define the causational relation between TAMs and NPC metastasis, NPC xenograft tumor-bearing mice were treated with clodronate liposomes to deplete TAMs. Expectedly, clodronate treatment ablated the total number of TAMs in 5-8F tumor tissues (Figure 7A). A significantly lower number of CD206^+^ TAMs was found in clodronate treated 5-8F tumor–bearing mice (Figure 7A). In *FGF2*-shRNA transfected group, clodronate further reduced the low level of macrophage infiltration (Figure 7A). Importantly, CTCs, tumor clones from blood culture, and pulmonary metastases were markedly inhibited in clodronate-treated 5-8F tumor–bearing mice (Figure 7, B and C, and Supplemental Figure 7, A and B), supporting TAM’s critical role in NPC pulmonary metastasis. To generalize these findings in FGF-2 expressing tumors, we treated FGF-2–overexpressing and control tumor–bearing mice with clodronate. Similarly, the control liposome did not significantly affect TAM infiltration, and the vector tumor group had a significantly lower number of TAMs in TME (Supplemental Figure 7C). FGF-2 tumor–bearing mice showed elevated CTC levels and increased tumor clones from blood culture, and approximately 80% of them developed pulmonary metastasis (Supplemental Figure 7, D–F, and Figure 7D). Vector tumor–bearing mice had lower levels of CTC and pulmonary metastasis (Supplemental Figure 7, D–F, and Figure 7D). The depletion of TAMs markedly decreased the metastasis rate of both vector and FGF-2 tumor–bearing mice (Supplemental Figure 7, D–F, and Figure 7D). These findings show that FGF-2–promoted pulmonary metastasis through a TAM-dependent mechanism.

## Discussion

Despite the increased need to understand the role of the TME in promoting tumor invasion and metastasis, key questions regarding how crosstalk between nontumor cell components contribute to tumor metastasis require further investigation. Particularly, vascular pericytes located between blood vessels and tumor cells may easily communicate with various cell types and become initiators of the metastatic cascade. The role of pericyte in the TME is diverse. As a major perivascular cell type in tumor microvessels, pericyte participates in angiogenesis and increases vessel maturation and stability, which support transportation of nutrients and oxygen for tumor growth. Additionally, pericyte coverage of microvessels impedes tumor cell intravasation. Recently, the inflammatory role of pericytes in the TME receives increasing attention. For example, pericyte-derived IL-33 promotes TAM infiltration (27). In malignant glioma, immature pericytes possess T cell inhibitory capability via expressing multiple immunosuppressive mediators (30). In primary CNS lymphoma, pericyte-derived CXCL9 and CXCL12 increase tumor-infiltrating lymphocytes, including CD8^+^ T cells (31). It seems that the inflammatory role of pericytes is context dependent. Moreover, pericytes may regulate the immune microenvironment through indirect mechanisms. For example, targeting pericytes induces leaky, dysfunctional microvessels, indirectly increasing hypoxia and resulting in myeloid-derived suppressor cell infiltration (32). Considering that pericytes’ phenotype varies with tumor types, and that pericyte closely communicates with other TME components, it is not difficult to understand that its inflammatory role is complex and somehow paradoxical. Indeed, although targeting pericytes has been proposed as a potential therapeutic option for treating solid tumors alone or together with antiangiogenic drugs (33–35), clinical trials blocking pericytes have failed to improve patients’ outcomes (36). We believe that in-depth studies of molecular mechanisms of pericyte-derived signaling molecules in the modulation of the TME will support us for understanding the complex role of tumoral pericytes and allow us to discover new therapeutic options.

In the current work, we have taken an unbiased approach to define pericyte-derived inflammatory signaling molecules upon FGF-2 challenge. We found that CXCL14 is highly upregulated and is a potent facilitator for TAM recruitment and polarization. Interestingly, although FGFRs are expressed at the same levels in both fibroblasts and pericytes, FGF-2–induced CXCL14 is exclusively expressed in pericytes, indicating that CXCL14 is one of the pericyte-specific inflammatory mediators. As a nonglutamic acid-leucine-arginine chemokine, CXCL14 has broad biological activities. It primarily contributes to the regulation of immune cell migration and also executes antimicrobial immunity. CXCL14 receptor remains an enigma, although recent research suggested that ACKR2 is required for CXCL14 signaling (24). It is reported that different CC or CXC chemokines can form heterodimers, and cross-family CC or CXC heterodimers have been reported (37, 38). That might explain the difficulties of CXCL14 receptor identification. Our work shows that CXCL14 promotes TAM recruitment and polarization, promoting tumor metastasis. These results are in line with clinical studies and the current knowledge of this chemokine (39). Notably, besides its inflammatory role, CXCL14 in the TME may directly stimulate malignant cells and contribute to EMT and tumor metastasis (24, 40). This role requires further validation in FGF-2–expressing tumors.

One of the striking findings is that, using a cross–data set approach, we identified NPC for expressing high levels of FGF-2. To our knowledge, this is the first time that NPC has been identified as a natural FGF-2–expressing human tumor type. The progress of molecular-targeted therapies in NPC significantly falls behind than that in other types of tumor, and several trials using bevacizumab or cetuximab in NPC treatment have failed to provide better clinical benefits than conventional therapies (5, 6). This work might provide mechanistic insights for the limited clinical outcomes of anti-VEGF and anti-EGFR treatments in NPC. It also offers potentially novel targets such as the FGF-2/CXCL14 axis for treating NPC. Of note, in addition to FGF-2 signaling, NPC may also affect pericytes through other angiogenic factors, such as angiopoietins. Whether these factors act together with FGF-2 on pericytes and metastasis has not been explored in depth and needs to be further investigated.

Moreover, NPCs are characterized by abundant infiltration of inflammatory cells, and several clinical trials have been conducted using immune checkpoint blockade therapies and show promising clinical activity (41, 42). Although the majority of immune cells in NPC are T lymphocytes, our results in NPC patient samples show that there is still a significant macrophage infiltration, which may be one of the major players for inducing metastasis. In other types of tumors, TAMs promote tumor progression by promoting genetic instability, nurturing cancer stem cells, supporting metastasis, and taming protective adaptive immunity; targeting TAMs by reducing or reprogramming them has shown promising activity in some clinical trials (43–45). However, in the NPC field, immunotherapy is mainly focused on T lymphocytes. Other types of immune cells, such as macrophages, have not been explored. Interestingly, a cohort of 108 NPC patients shows that the expression of macrophage inhibitory factor, a highly conserved cytokine that inhibits macrophage migration, can independently predict the survival of NPC patients (46), indicating that macrophages might be involved in NPC progression or metastasis. In our work, macrophage-depleting reagent clodronate liposomes significantly reduce NPC metastasis. Notably, due to the lack of spontaneous mouse NPC models or mouse NPC cell lines, we exploited other types of mouse tumor for FGF-2 overexpression experiments and genetically modified mouse model experiments. These experiments cannot fully recapitulate the characteristics of human NPC — rather, they illustrate the generalized mechanism of FGF-2 in various tumors. Although our present work is originated from and focused on NPC, we believe that these mechanistic principles may also apply to other solid cancers that express FGF-2. Our results suggest that targeting TAMs would be a potent antimetastasis therapy in NPC or other FGF-2–expressing tumors.

**Figure 8:**
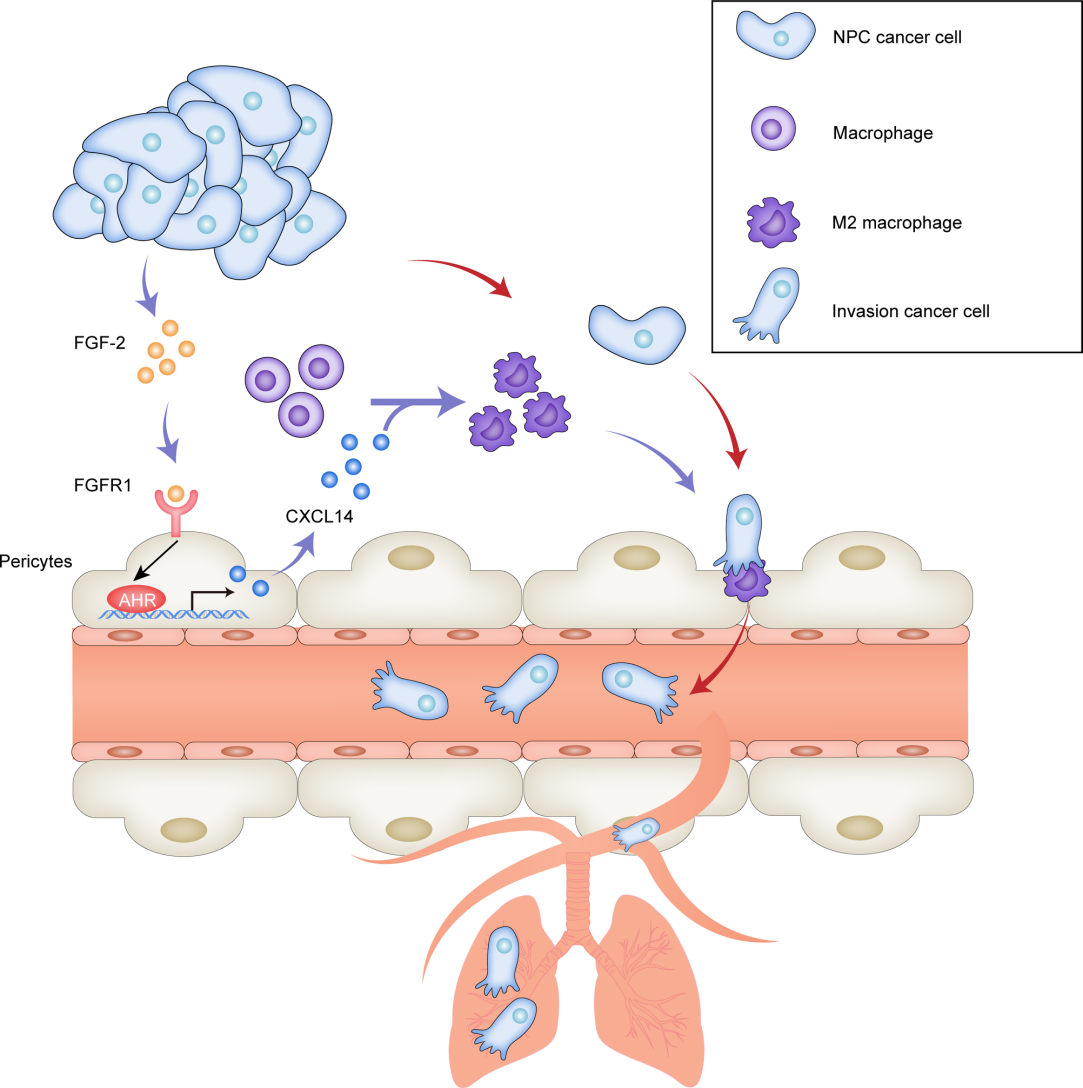
Schematic diagram of pericyte-associated FGF-2/FGFR1/AHR/CXCL14 axis recruits and polarizes TAMs in facilitating NPC metastasis. (**A**) NPC cancer cells often produce FGF-2, and FGF-2 primarily targets pericytes and fibroblasts. In FGF-2^+^ tumors, vascular-associated pericytes and CAFs express various inflammatory regulating cytokine/chemokines. Among them, CXCL14 is produced exclusively by pericytes through FGF-2/FGFR1/AHR signaling. CXCL14 signaling recruits and polarizes TAMs into an M2-like phenotype. M2-like TAMs facilitate tumor cell intravasation and pulmonary metastasis.

To our knowledge, this is the first study to investigate the role of pericytes in the field of NPC. Our work provides an example of malignant cell–orchestrated stromal cell–stromal cell interactions in facilitating cancer metastasis (Figure 8). NPC cell–derived FGF-2 educates the vascular-associated pericytes for producing CXCL14. Additionally, CXCL14-recruited/polarized M2 TAMs facilitate cancer cell intravasation and metastasis. Thus, NPC cells, or FGF-2–expressing malignant cells, orchestrate these 2 cellular components in the tumor stroma to promote metastasis. These findings provide mechanistic insights into NPC metastasis and define therapeutic targets in the FGF-2/FGFR1/pericyte/CXCL14/TAMs axis for treating metastasis of NPC or FGF-2–expressing malignancies.

## Methods

Supplemental Methods are available online with this article.

### Cell culture.

Human 5-8F NPC and SUNE-1 NPC cell lines were provided by Zesong Li at Shenzhen Second People’s Hospital, The First Affiliated Hospital of Shenzhen University. Murine RAW 264.7 monocyte and human THP-1 monocyte cell lines were provided by Dapeng Yan at the School of Basic Medical Sciences, Fudan University. Human A549 lung carcinoma cell line was kindly provided by Yongbo Wang at the School of Basic Medical Sciences, Fudan University. Murine T241 fibrosarcoma, murine MS5 stromal fibroblasts, murine 4T1 breast cancer, human SK-MEL-5 melanoma, human Hep3B hepatocellular carcinoma, human MCF-7 breast cancer, human A-431 squamous carcinoma, and human 293T embryonic kidney cell lines were provided by Yihai Cao at the Karolinska Institutet. The human TERT-immortalized fibroblast cell line was provided by George Klein at the Karolinska Institutet. Human primary pericytes were provided by Dongmei Zhang at the College of Pharmacy, Jinan University (47). Murine primary endothelial cells and primary pericytes were isolated from healthy mice by FACS. Human HUVEC endothelial cells were purchased from ATCC. Human FGF-2 and control vector were expressed at high levels with GFP in T241 and 4T1 cell lines (35). Sh*Scrambled* vector and sh*FGF2* vector were transfected into 5-8F cell lines with GFP using a lentiviral system (GeneCopoeia Inc.). THP-1, A549, SUNE-1, and 5-8F cell lines were cultured in 10% FBS-RPMI 1640 (catalog 40130ES76, YEASEN; catalog MA0215, Meilunbio), containing 100 U/mL penicillin and 100 μg/mL streptomycin (catalog MA0110, Meilunbio). RAW 264.7, T241, 4T1, MS5, mouse pericytes, SK-MEL-5, Hep3B, MCF-7, A-431, and 293T cell lines were cultured in 10% FBS-DMEM (catalog 40130ES76, YEASEN; catalog MA0213, Meilunbio), containing 100 U/mL penicillin and 100 μg/mL streptomycin (catalog MA0110, Meilunbio). Human pericytes were cultured in Pericyte Medium (catalog 1201, ScienCell). HUVEC and mouse endothelial cells were cultured in 10% FBS-M199 (catalog 40130ES76, YEASEN; catalog SH30253.01, HyClone), containing 100 U/mL penicillin and 100 μg/mL streptomycin (catalog MA0110, Meilunbio). All cell lines used in our study were negative for mycoplasma using a PCR method with 2 primer pairs: forward: 5′-GGCGAATGGGTGAGTAACACG-3′ and reverse: 5′-CGGATAACGCTTGCGACCTATG-3′; forward: 5′-GGGAGCAAACAGGATTAGATACCCT-3′ and reverse: 5′-TGCACCATCTGTCACTCTGTTAACCTC-3′.

### Cell isolation.

Fresh tissues were cut into small pieces in ice-cold PBS, and then digested in PBS containing 0.1% collagenase I and II (catalog 40507ES60, YEASEN; catalog 40508ES60, YEASEN) in 37°C for 30 minutes with gentle pipetting. After digestion, cells were washed with ice-cold PBS and resuspended by 1 mL MACS buffer (a solution containing PBS, 0.5% BSA, and 2 mM EDTA) and stained with an Alexa Fluor 647–conjugated anti–mouse F4/80 antibody (catalog 12322, BioLegend) or a rabbit anti–mouse NG2 antibody (catalog AB5320, Millipore), followed by an Alexa Fluor 647–conjugated donkey anti-rabbit antibody (catalog A31573, Invitrogen). Anti–Alexa Fluor 647 MicroBeads (catalog 130-091-395, Miltenyi Biotec; catalog 130-042-303, Miltenyi Biotec) were subsequently used for magnetic labelling. After washing, positive and negative cells were sorted with a MACS column and magnetic MACS separators (catalog 130-042-201, Miltenyi Biotec). NG2^+^ and NG2^–^ populations were collected for following experiments.

### Animals.

Female C57BL/6 and BALB/c-nude mice at the age between 6 and 8 weeks old were purchased from GemPharmatech, and they were maintained under a 12-hour dark/12-hour light cycle with food and water provided ad libitum. C.FVB-Tg(Cspg4-TK*)1Rkl/J (NG2-tk on BALB/c) mice were provided by Raghu Kalluri at the Metastasis Research Center, University of Texas MD Anderson Cancer Center, Houston, Texas, USA. All animals were randomly assigned to groups before experiments. The experimenter was not blind to the assignment of the groups and the evaluation of the results. No samples, animals, or data were excluded.

### Human patient samples.

Fresh samples were collected from patients receiving nasopharyngoscopic biopsy. Human NPC cancer cells and noncancer cells were isolated from fresh NPC tissues using a human tumor cell isolation kit (catalog 130-108-339, Miltenyi Biotec).

### Tumor metastasis models.

Approximately 1 × 10^6^ T241-vector or T241-FGF-2 tumor cells in 50 μL PBS (catalog MA0015, Meilunbio) were s.c. implanted into each C57BL/6 mouse. A total of 1 × 10^6^ 5-8F sh*Scrambled* or 5-8F sh*FGF2* tumor cells in 50 μL PBS were s.c. implanted into each BALB/c-nude mice. For 4T1 orthotopic tumor models, 1 × 10^6^ 4T1-vector or 4T1–FGF-2 tumor cells in 50 μL PBS mixed with 50 μL Matrigel Matrix (catalog 354234, Corning) were injected into the mammary fat pad of female C.FVB-Tg(Cspg4-TK*)1Rkl/J mice. Tumor sizes were measured every other day with a calliper, and tumor volumes were calculated according to a standard formula: Tumor volume = length × width^2^ × 0.52 (11). Tumor removal was surgically performed under anesthesia, when primary tumor volumes reached the size of 2.0–2.5 cm^3^. Mice were kept for an additional 4–6 weeks for metastasis detection. GFP^+^ metastatic nodules were detected by an IVIS system (VISQUE Invivo Elite, VIEWORKS). Lung tissues were subsequently paraffin embedded, stained with H&E, and examined under light microscopy.

### Histological analysis, IHC, and immunofluorescence.

For histological analysis, tumor or lung tissues were fixed with 4% paraformaldehyde (PFA) (catalog MA0192, Meilunbio) for 12 hours at room temperature. Paraffin-embedded tissues were cut into the thickness of 5 μm, mounted onto glass slides, baked for 1 hour at 60°C, deparaffinized in Xylene (catalog 10023418, Sinopharm Chemical Reagent Co. [SCR]), and sequentially rehydrated in 99%, 95%, and 70% ethanol (catalog 10009218, SCR). Tissue slides were counterstained with H&E (catalogs MB9897 and MA0164, Meilunbio) before dehydration with 95% and 99% ethanol, and they were mounted with neutral balsam (catalog 1004160, SCR). Stained tissues were analyzed under a light microscope (Leica DM IL LED). For IHC staining of tumor tissues, paraffin-embedded tissue sections were stained with a rabbit anti–FGF-2 antibody (catalog A0235, ABclonal, 1:100); a mouse anti-FSP1 antibody (catalog 66489-1, Proteintech, 1:100); a rabbit anti-CD163 antibody (catalog A8383, ABclonal, 1:100); a rabbit anti-F4/80 antibody (catalog 70076, Cell Signaling Technology, 1:1000); and a goat anti-CD206 antibody (catalog AF2535, R&D system, 1:400). After rinsing, tissue samples were further stained by IHC secondary antibodies, an anti-rabbit IgG (HRP) antibody (catalog ab205718, Abcam), or an anti–goat IgG (HRP) antibody (catalog A21030, Abbkine). Positive signals were captured using a microscope (catalog DM2500, Leica). For immunofluorescence double staining, similar to our previous work (48, 49), paraffin-embedded tumor tissue sections were stained with a rabbit anti-CD31 antibody (catalog ab182981, Abcam, 1:1000). After rinsing, tissue samples were further stained for 45 minutes at 37°C with a secondary antibody, HRP-conjugated goat anti–rabbit IgG (catalog ab205718, Abcam, 1:4000). Alexa Fluor 555 Tyramide Super Boost Kit (catalog B40923, Thermo Fisher Scientific) was used for antigen visualization. Next, tumor tissue sections were stained again with a rabbit anti-NG2 antibody (catalog AB5320, MilliporeSigma, 1:200) and then stained with a donkey anti–rabbit Alexa Fluor 488 antibody (catalog A21206, Invitrogen, 1:4000). Slides were mounted with antifading mounting medium (with DAPI) (catalog MA0236, Meilunbio). Positive signals were captured using a fluorescence microscope (Olympus BX53). Captured images were further analyzed using the Adobe Photoshop CS software.

### RNA extraction and qPCR.

Total RNAs were extracted from various tissues and cultured cells using an RNAsimple Total RNA kit (catalog DP419, TIANGEN). Total RNA from each sample was reversely transcribed using a Hifair II 1st Strand cDNA Synthesis SuperMix (catalog 11123ES60, YEASEN). Reverse transcription was performed at 42°C for 30 minutes and subsequently at 85°C for 5 minutes to inactivate the enzyme activity. The cDNA samples were subjected to qPCR using a StepOnePlus Real-Time PCR System (Applied Biosystems). Each sample was triplicated and in a 10 μL reaction containing Hieff qPCR SYBR Green Master Mix (catalog 11203ES03, YEASEN), 50 nM forward and reverse primers, and 2 μL cDNA. The qPCR protocol was executed for 40 cycles, and each cycle consisted of denaturation at 95°C for 15 seconds, annealing at 60°C for 1 minute, and extension at 72°C for 1 minute. The primer pairs specific for various genes used in our experiments included: human *FGF2* forward: 5′-AGAAGAGCGACCCTCACATCA-3′; human *FGF2* reverse: 5′-CGGTTAGCACACACTCCTTTG-3′; human *CD163* forward: 5′-TTTGTCAACTTGAGTCCCTTCAC-3′; human *CD163* reverse: 5′-TCCCGCTACACTTGTTTTCAC-3′; human *CD31* forward: 5′-AACAGTGTTGACATGAAGAGCC-3′; human *CD31* reverse: 5′-TGTAAAACAGCACGTCATCCTT-3′; human *NG2* forward: 5′-CTTTGACCCTGACTATGTTGGC-3′; human *NG2* reverse: 5′-TGCAGGCGTCCAGAGTAGA-3′; human *FSP1* forward: 5′-GATGAGCAACTTGGACAGCAA-3′; human *FSP1* reverse: 5′-CTGGGCTGCTTATCTGGGAAG-3′; human *FGFR1* forward: 5′-CCCGTAGCTCCATATTGGACA-3′; human *FGFR1* reverse: 5′-TTTGCCATTTTTCAACCAGCG-3′; human *FGFR2* forward: 5′-AGCACCATACTGGACCAACAC-3′; human *FGFR2* reverse: 5′-GGCAGCGAAACTTGACAGTG-3′; human *FGFR3* forward: 5′-TGCGTCGTGGAGAACAAGTTT-3′; human *FGFR3* reverse: 5′-GCACGGTAACGTAGGGTGTG-3′; human *FGFR4* forward: 5′-GAGGGGCCGCCTAGAGATT-3′; human *FGFR4* reverse: 5′-CAGGACGATCATGGAGCCT-3′; human *CD206* forward: 5′-TCCGGGTGCTGTTCTCCTA-3′; human *CD206* reverse: 5′-CCAGTCTGTTTTTGATGGCACT-3′; human *CD86* forward: 5′-CTGCTCATCTATACACGGTTACC-3′; human *CD86* reverse: 5′-GGAAACGTCGTACAGTTCTGTG-3′; human *CXCL14* forward: 5′-CGCTACAGCGACGTGAAGAA-3′; human *CXCL14* reverse: 5′-GTTCCAGGCGTTGTACCAC-3′; human *GAPDH* forward: 5′-CTGGGCTACACTGAGCACC-3′; human *GAPDH* reverse: 5′-AAGTGGTCGTTGAGGGCAATG-3′; mouse *Fgf2* forward: 5′-TGGTGACCACAAGCTGAATG-3′; mouse *Fgf2* reverse: 5′-TCCCTTGATAGACACAACTCCTC-3′; mouse *Fgfr1* forward: 5′-TAATACCACCGACAAGGAAATGG-3′; mouse *Fgfr1* reverse: 5′-TGATGGGAGAGTCCGATAGAGT-3′; mouse *Fgfr2* forward: 5′-CCTCGATGTCGTTGAACGGTC-3′; mouse *Fgfr2* reverse: 5′-CAGCATCCATCTCCGTCACA-3′; mouse *Fgfr3* forward: 5′-GCCTGCGTGCTAGTGTTCT-3′; mouse *Fgfr3* reverse: 5′-TACCATCCTTAGCCCAGACCG-3′; mouse *Fgfr4* forward: 5′-GCTCGGAGGTAGAGGTCTTGT-3′; mouse *Fgfr4* reverse: 5′-CCACGCTGACTGGTAGGAA-3′; mouse *Ccl11* forward: 5′-GAATCACCAACAACAGATGCAC-3′; mouse *Ccl11* reverse: 5′-ATCCTGGACCCACTTCTTCTT-3′; mouse *Cxcl14* forward: 5′-GAAGATGGTTATCGTCACCACC-3′; mouse *Cxcl14* reverse: 5′-CGTTCCAGGCATTGTACCACT-3′; mouse *Cd206* forward: 5′-CTCTGTTCAGCTATTGGACGC-3′; mouse *Cd206* reverse: 5′-CGGAATTTCTGGGATTCAGCTTC-3′; mouse *Cd86* forward: 5′-TGTTTCCGTGGAGACGCAAG-3′; mouse *Cd86* reverse: 5′-TTGAGCCTTTGTAAATGGGCA-3′; mouse *Ahr* forward: 5′-AGCCGGTGCAGAAAACAGTAA-3′; mouse *Ahr* reverse: 5′-AGGCGGTCTAACTCTGTGTTC-3′; mouse *Gapdh* forward: 5′-AGGTCGGTGTGAACGGATTTG-3′; and mouse *Gapdh* reverse: 5′-TGTAGACCATGTAGTTGAGGTCA-3′.

### Immunoblot.

Cultured cells were lysed in a RIPA lysis buffer containing proteinase and phosphatase inhibitor cocktails (catalog MA0151, Meilunbio; catalog MB2678, Meilunbio, 1:100). An equal amount of protein samples from each group and a standard molecular weight marker (catalog AP13L052, Life-iLab) were loaded on a 10% SDS-PAGE gel (catalog AP15L945, Life-iLab), followed by transferring onto a polyvinylidene difluoride (PVDF) membrane (catalog IPVH00010, MilliporeSigma), which was subsequently blocked with 5% skimmed milk for 2 hours. Membranes were incubated overnight at 4°C with primary antibodies diluted in a Primary Antibody Dilution Buffer (catalog MB9881, Meilunbio). After rigorous washing with PBS containing 0.1% Tween-20 (catalog T8220, Solarbio), membranes were incubated at room temperature for 1 hour with a goat anti–mouse HRP–conjugated IgG antibody (catalog AS003, ABclonal, 1:5000) or a goat anti–rabbit HRP–conjugated IgG antibody (catalog AS014, ABclonal, 1:5000). Target proteins were visualized via a super sensitive ECL luminescence reagent (catalog MA0186, Meilunbio) with a Molecular Imager ChemiDoc XRS System (Bio-Rad). A rabbit anti–β-tubulin antibody (catalog ab6046, Abcam, 1:5000), a rabbit anti-AKT (pan) antibody (catalog 4691, Cell Signaling Technology, 1:2000), a mouse anti–phospho-AKT antibody (catalog 66444-1, Proteintech, 1:1000), a rabbit anti-ERK1/2 (catalog 4695, Cell Signaling Technology, 1: 2000), a rabbit anti–phospho-ERK1/2 (catalog 4370, Cell Signaling Technology, 1:2000), a rabbit anti-CD206 antibody (catalog 141702, BioLegend, 1:2000), a rabbit anti-CD86 antibody (catalog BM4121, BOSTER, 1:1000), and a mouse anti–β-actin antibody (catalog AC004, Abclonal, 1:2000) were used as primary antibodies.

### ChIP.

ChIP assay was performed using a ChIP assay kit (catalog p2078, Beyotime). DNA-bound proteins were fixed using 4% PFA. Chromatin was purified and sonicated to generate fragments of approximate sizes between 500 and 1000 bp. In total, 20 μL of the sonicated chromatin was collected for input. A total of 180 μL of the sonicated chromatin was immunoprecipitated by a rabbit anti-AHR antibody (catalog NB100-2289, Novus, 1:200) and a rabbit nonimmune IgG antibody (catalog AC005, ABclonal, 1:200). The protein-DNA complexes were mixed with 5M NaCl and incubated at 65°C for 4 hours. The purified DNA fraction was used for qPCR analysis. The mouse *Cxcl14* promoter primer pair includes the following: forward 5′-TGGACCACGAGCCCAGCAAG-3′, reverse 5′-TTTACTGTCCGAAGCCACCG-3′. The mouse *Cxcl14* exon 3 primer pair includes the following: forward 5′-AGAAGATGGTTATCATCACC-3′, reverse 5′-TTCTTCGTAGACCCTGCGCT-3′. Data were normalized with the non–immune rabbit IgG values.

### Cell viability assay.

Cell viability was evaluated using a cell counting kit-8 (catalog MA0218, Meilunbio) following the manufacturer’s protocol. Cells were seeded into 96-well plates at a density of ~5000 cells/well and incubated until various time points. Subsequently, 10 μL of the solution containing 2-(2-methoxy-4-nitrophenyl)-3-(4-nitrophenyl)-5-(2,4-disulfophenyl)-2H-tetrazolium (WST-8) was added, and the cells were cultured at 37°C for 1 hour. OD values were measured at 450 nm via a Synergy 2 Multi-Mode Microplate Reader (BioTek).

### Wound healing.

Wound healing experiments were performed to test the migrating ability of the cell. Cells were seeded in 6-well plates with a density of approximately 1 × 10^6^ cells/well. For coculture models, GFP^+^ cancer cells were seeded with approximately 7.5 × 10^5^ cells/well, whereas fibroblast, pericytes, endothelial cells, or macrophages were seeded with approximately 2.5 × 10^5^ cells/well. Wounds were created by scraping monolayer cells, and nonadherent cells were washed off. Cells were incubated in 1% FBS medium for another 24 hours at 37°C. GFP^+^ signals were captured by a fluorescent microscope (catalog EVOS M5000, Thermo Fisher Scientific). Cell migration was determined by measuring the width of the scratched area. ImageJ software (Version 2.1.4.7, NIH, USA) was used to quantify the scratched area.

### Chemotaxis assay.

Cells were seeded into a transwell chamber (catalog 725301, NEST) containing pores of 8 μm in diameter at an initial seeding density of 1 × 10^5^ cells/well. The transwell chambers were then inserted into the wells of a 24-well plate with or without CXCL14 (catalog 730-XC-025, R&D systems, 100 ng/mL) and were kept at 37°C for another 24 hours. The migrated cells were stained with crystal violet (catalog MA0149, Meilunbio), and the number of migratory cells was recorded using an optical microscope (catalog DM2500, Leica).

### FACS analysis.

For CTC detection, after sacrificing the tumor-bearing animals, peripheral blood was collected and transferred into an anticoagulation tube. RBC lysis buffer (catalog MA0207, Meilunbio) was used to remove RBCs at room temperature for 2 minutes. Cells were then washed 2 times with PBS. GFP^+^ CTCs were detected using a FACS system (FACSCanto II, BD Biosciences), whereas healthy mouse blood and in vitro–cultured GFP^+^ tumor cells were used as controls. FlowJo software (version 10, BD Biosciences) was used to analyze the FACS result. For tumor immune microenvironment analysis, tumor tissues were dissected and homogenized. Tissue suspension was treated for 3 minutes with 5 mL of an RBC lysis buffer (catalog MA0207, Meilunbio). After PBS washing, cell suspension was fixed for 30 minutes with 4% PFA. Single-cell suspensions were incubated with an eFluor 780 Viability Dye (catalog 65-0865-14, eBioscience) for 10 minutes and were incubated with various conjugated antibodies for 30 min on ice. These antibodies include: an eFluor 506 anti–mouse CD45 antibody (catalog 69-0451-82, eBioscience); a PE-Cyanine7 anti–mouse CD11b antibody (catalog 25-0112-82, eBioscience); an eFluor 450 anti–mouse Ly-6G antibody (catalog 48-9668-82, eBioscience); a PerCP-Cyanine5.5 anti–mouse MHC Class II antibody (catalog 65-5321, Tonbo Biosciences); a PE anti–mouse CD11c antibody (catalog 12-0114-82, eBioscience); an APC anti–mouse F4/80 antibody (catalog 17-4801-82, eBioscience); a PE anti–mouse CD206 antibody (catalog 141706, BioLegend); an eFluor 450 anti–mouse CD86 antibody (catalog 48-0862-82, eBioscience); an APC anti–mouse CD49b antibody (catalog 17-5971-82, eBioscience); a PE anti–mouse B220 antibody (catalog 12-0452-82, eBioscience); an eFluor 450 anti–mouse CD3e antibody (catalog 48-0031-82, eBioscience); and an APC anti–mouse Ly-6C antibody (catalog 17-5932-82, eBioscience). The stained cells were applied onto FACScan (BD Biosciences) and analyzed by Flowjo software (version 10, BD Biosciences).

### Drug treatment.

For pericytes depletion, NG2-tk and WT mice received daily i.p. injections with 50 mg/kg of ganciclovir for 3 days, starting from average tumor size reached 0.5 cm^3^. Ganciclovir was then given every third day to maintain a full depletion of pericytes. Tumor tissues were collected when tumor size reached 2 cm^3^ for checking pericyte depletion efficacy. For CXCL14 in vivo administration, 0.5 μg recombinant human CXCL14 protein (catalog 730-XC-025, R&D systems) was intratumorally injected by a 31 gauge needle in 20 μL volume on a daily basis for 5 days. Tumor tissues were collected for further investigation. For deleting TAMs, clodronate liposomes and control (catalog 40337ES10, YEASEN) at 30 mg/kg was i.p. injected once per week to each mouse until the tumor was removed. Tumor tissues were collected for TAMs infiltration detection. For in vitro experiments, cells were starved overnight with 1% FBS-DMEM, followed by the treatment of an FGFR1 inhibitor P173074 (catalog HY-10321, MedChemExpress), an FGFR2 inhibitor Alofanib (catalog S8754, Selleck), an FGFR4 inhibitor FGF401 (catalog S2801, Selleck), an FGFR pan-inhibitor AZD4547 (catalog S2801, Selleck), a pan-AKT kinase inhibitor AZD5363 (catalog S8019, Selleck), an ERK1/2 inhibitor SCH772984 (catalog S2801S7101, Selleck), or a MEK1/2 inhibitor U0126 (HY-12031A, MedChemExpress), with or without 100 ng/mL FGF-2 (catalog 10014-HNAE, SinoBiological). Forty-eight hours later, the cell lysates were collected for qPCR or Western blot analysis. CXCL14 (catalog 730-XC-025, R&D systems) at 100 ng/mL was used to stimulate RAW 264.7 monocytes. DMSO (catalog MB2505, Meilunbio) or PBS was used as a control. For conditioned medium collection, cells were starved overnight with 1% FBS-DMEM, followed by a 100 ng/mL FGF-2 treatment for 48 hours; PBS was used as a control.

### Microarrays and RNA-Seq analysis.

Gene expression profiles and clinical information of TCGA pan-cancer data were downloaded from the UCSC Xena database (https://xenabrowser.net/datapages/). Affymetrix Human Genome U133 Plus 2.0 Array data with accession no. GSE12452 was downloaded from the GEO. R package “ggplot2” was used to perform differentially expressed genes analysis. RNA expression levels of selected genes were presented as log_2_ ratio, and heatmaps were made by GraphPad Prism. For transcription factor prediction, a transcription factor prediction tool, PROMO (http://alggen.lsi.upc.es/cgi-bin/promo_v3/promo/promoinit.cgi?dirDB=TF_8.3), was used. A 2000 bp promoter of *Cxcl14* was analyzed. For the pericyte genome-wide microarray, NG2^+^ pericytes from T241–FGF-2–expressing tumors or vector tumors were isolated by FACS. Briefly, fresh tumor samples were cut into small fractions and digested in 0.15% collagenase I and II containing PBS solution at 37°C for 40 minutes. After PBS wash, the cell suspension was incubated with an anti-NG2 antibody (catalog AB5320, MilliporeSigma) for 45 minutes on ice, followed by a goat anti-rabbit secondary antibody conjugated with Cy3 (catalog A10520, Invitrogen) for 30 minutes on ice. NG2^+^EGFP^–^ population from 3 individual biological samples was sorted by flow cytometry (MoFlo XTD, Beckman Coulter). Isolated cells were stored in RNA later (catalog R0901, Sigma-Aldrich) until further analysis. Microarray hybridization, scanning, normalization, and analysis were accomplished by Shanghai Biotechnology Corporation using Affymetrix 2.0 ST mouse gene arrays. The data were presented as heatmaps and volcano plots, with *P* values and fold changes applying to all genes in the chemokine family available in the data sets. Gene array data were deposited in the GEO with an accession no. GSE197794.

### Statistics.

Statistical computations were performed using GraphPad Prism (GraphPad). The data were found to pass the D’Agostino-Pearson normality test. Statistical differences between 2 groups were determined by a 2-tailed Student’s *t* test. *P <* 0.05 was considered statistically significant, *P <* 0.01 was very significant, and *P <* 0.001 was extremely significant. Differences among multiple groups were evaluated using 1-way ANOVA. The data are presented as mean ± SD.

### Study approval.

All animal studies were approved by the Animal Experimental Ethical Committee of the Fudan University (no. 20200306-071). All human studies were approved by the Ethical Review Committee in the Shenzhen Second People’s Hospital, Shenzhen, China (no. 20200525002).

## Author contributions

Y Yang and KH generated the ideas and designed experiments. YW, QS, and Y Ye performed most experiments and organized all figures. XS, SX, YZ, JS, XF, BZ, and MY were involved in some of the experiments. LL, KH, and GN provided important reagents and participated in discussions. Y Yang wrote the manuscript. The authorship order among co–first authors was determined by relative contribution.

## Supplementary Material

Supplemental data

## Version 1 04/19/2022

In-Press Preview

## Version 2 05/23/2022

Electronic publication
