# Supplementary material for: FGF-2 signaling in nasopharyngeal carcinoma modulates pericyte-macrophage crosstalk and metastasis
Source: JCI Insight. 2022 May 23;7(10):e157874. doi: 10.1172/jci.insight.157874 (PMC9220856; doi:10.1172/jci.insight.157874)
Supplement: Supplemental data [file jciinsight-7-157874-s233.pdf]

Supplemental Information

FGF-2 signaling in nasopharyngeal carcinoma modulates pericyte-macrophage crosstalk and metastasis

Yujie Wang[1#], Qi Sun[2#], Ying Ye[3#], Xiaoting Sun[4], Sisi Xie[2], Yuhang Zhan[2], Jian Song[1], Xiaoqin Fan[1], Bin Zhang[1], Ming Yang[5], Lei Lv[6], Kayoko Hosaka[7*], Yunlong Yang[1,2*], and Guohui Nie[1,8*]

[1]Shenzhen Key Laboratory of nanozymes and Translational Cancer Research, Department of Otolaryngology, Shenzhen Institute of Translational Medicine, The First Affiliated Hospital of Shenzhen University, Shenzhen Second People's Hospital, Shenzhen 518035, China

[2]Department of Cellular and Genetic Medicine, School of Basic Medical Sciences; Fudan University, Shanghai 200032, China

[3]Department of Oral Implantology, School and Hospital of Stomatology, Tongji University; Shanghai Engineering Research Center of Tooth Restoration and Regeneration, Shanghai, China

[4]Department of Medical Oncology, Shuguang Hospital, Shanghai University of Traditional Chinese Medicine, 201203 Shanghai, China

[5]Department of Otolaryngology, Shenzhen People's Hospital, the Second Clinical Medical College, Jinan University, the First Affiliated Hospital, Southern University of Science and Technology, Shenzhen 518020, Guangdong, China

[6]Ministry of Education Key Laboratory of Metabolism and Molecular Medicine, Department of Biochemistry and Molecular Biology, School of Basic Medical Sciences, Fudan University, Shanghai 200032, China

[7]Department of Microbiology, Tumor and Cell Biology, Karolinska Institutet, 171 77 Stockholm, Sweden

[8]State Key Laboratory of Chemical Oncogenomics, Guangdong Provincial Key Laboratory of Chemical Genomics, Peking University Shenzhen Graduate School, Shenzhen 518055, China

Keywords: fibroblast growth factor-2, nasopharyngeal carcinoma, metastasis, tumor-associated macrophage, chemokine (C-X-C motif) ligand 14, pericytes

[#]These authors contributed equally.
***Corresponding authors.** Galley proofs and reprint requests should be addressed to: Kayoko Hosaka, M.D, Ph.D., Department of Microbiology, Tumor and Cell Biology, Karolinska Institutet, Stockholm, Sweden. Tel: (+46)-852486299, Email: kayoko.hosaka@ki.se; Yunlong Yang, Ph.D., Department of Cellular and Genetic Medicine, School of Basic Medical Sciences, Fudan University, Shanghai 200032, P.R. China. Tel: (+86)-21-54237311, E-mail: yunlongyang@fudan.edu.cn; Guohui Nie, M.D, Department of ENT, Second People's Hospital of Shenzhen, First Affiliated Hospital of Shenzhen University, Shenzhen 518035. Tel: (+86)-755-83366388, Email: nieguohui@email.szu.edu.cn;

Running title: FGF-2 promotes metastasis via pericyte-derived CXCL14

Supplemental Methods

Enzyme-linked immunosorbent assay

ELISA was used for measuring FGF-2 protein concentrations. Cultured cells were homogenized in a RIPA buffer with the proteinase and phosphatase inhibitor cocktail (Cat. No. MA0151, Meilunbio, China; Cat. No. MB2678, Meilunbio, China; 1:100), followed by 15-minute centrifugation. Supernatants were stored at–80°C until further analysis. A human basic fibroblast growth factor, bFGF ELISA Kit (Cat. No. CSB-E08000h, Cusabio Technology) was performed according to the manufacturer’s protocol using the appropriate standard curve. Absorbance values were detected at 450 nm using a microplate reader (Cat. No. SPARK 10M, TECAN).

Blood culture

Peripheral blood of tumor-bearing mice was collected and transferred to an anti-coagulation tube. RBC lysis buffer (Cat. No. MA0207, Meilunbio, China) was used to remove red blood cells at room temperature for 2 min. Cells were then washed 2 times with PBS. The cell suspension was seeded onto 6-well plates for 24 h and the non-adherent cells were removed by changing the medium. Cells were cultured with 10% FBS-DMEM (Cat. No. 10099-141, Gibco; Cat. No. MA0213, Meilunbio, China) for 10 days, and stained with crystal violet (Cat. No. MA0149, Meilunbio, China) for further analysis.

SiRNA and shRNA knockdown

Ahr siRNA and scrambled control siRNA were purchased (GenePharma, China) and transfected into mouse primary pericytes using liposomal transfection reagent (Cat. No.

40802ES03, YEASEN, China). The knockdown efficiency was detected by qPCR after 48 h. For shRNA experiments, sh*Scramble* vector and sh*FGF2* vector were transfected into 5-8F cell line with GFP using a lentiviral system (GeneCopoeia Inc., USA). Briefly, 293T cells were transfected with lentiviral vectors for lentivirus production. Cells were cultured for an additional 72 h, and the conditioned medium containing lentiviral particles were used for stably transfecting 5-8F NPC cell lines with polybrene (Cat. No. 40804ES76, YEASEN, China). Puromycin (Cat. No. 60210ES25, YEASEN, China) was used for selecting the successfully transfected NPC clones. The knockdown efficacy was detected by qPCR.

Supplemental Tables

Tissue type	Gender	Age	Location	Histopathology	Used in
NPC	Female	21	Nasopharynx	Undifferentiated non-keratinizing carcinoma	Fig. 1D, F
NPC	Male	31	Nasopharynx	Differentiated non-keratinizing carcinoma	
NPC	Male	37	Nasopharynx	Undifferentiated non-keratinizing carcinoma	
NPC	Female	45	Nasopharynx	Undifferentiated non-keratinizing carcinoma	
NPC	Male	41	Nasopharynx	Undifferentiated non-keratinizing carcinoma	
NPC	Male	65	Nasopharynx	Undifferentiated non-keratinizing carcinoma	
NPC	Male	41	Nasopharynx	Undifferentiated non-keratinizing carcinoma	Fig. 1E
NPC	Female	55	Nasopharynx	Undifferentiated non-keratinizing carcinoma	
NPC	Male	41	Nasopharynx	Undifferentiated non-keratinizing carcinoma	
NPC	Female	54	Nasopharynx	Undifferentiated non-keratinizing carcinoma	Fig. 1G
NPC	Female	24	Nasopharynx	Undifferentiated non-keratinizing carcinoma	
NPC	Male	46	Nasopharynx	Differentiated non-keratinizing carcinoma	
NPC	Male	54	Nasopharynx	Undifferentiated non-keratinizing carcinoma	
NPC	Male	46	Nasopharynx	Undifferentiated non-keratinizing carcinoma	
NPC	Male	41	Nasopharynx	Differentiated non-keratinizing carcinoma	
Rhinitis	Male	22	Nasopharynx	Chronic nasopharyngeal mucositis	Fig. 1D and F
Rhinitis	Female	25	Nasopharynx	Chronic nasopharyngeal mucositis	
Rhinitis	Male	51	Nasopharynx	Chronic nasopharyngeal mucositis	
Rhinitis	Female	60	Nasopharynx	Chronic nasopharyngeal mucositis	
Rhinitis	Female	33	Nasopharynx	Chronic nasopharyngeal mucositis	
Rhinitis	Female	38	Nasopharynx	Chronic nasopharyngeal mucositis	
Rhinitis	Male	43	Nasopharynx	Chronic nasopharyngeal mucositis	
Rhinitis	Female	27	Nasopharynx	Chronic nasopharyngeal mucositis	

Rhinitis	Male	27	Nasopharynx	Chronic nasopharyngeal mucositis	
Rhinitis	Male	15	Nasopharynx	Chronic nasopharyngeal mucositis	
Rhinitis	Female	9	Nasopharynx	Chronic nasopharyngeal mucositis	
Rhinitis	Male	8	Nasopharynx	Chronic nasopharyngeal mucositis	
Rhinitis	Male	5	Nasopharynx	Chronic nasopharyngeal mucositis	Fig. 1G
Rhinitis	Male	12	Nasopharynx	Chronic nasopharyngeal mucositis	
Rhinitis	Male	25	Nasopharynx	Chronic nasopharyngeal mucositis	

Table S1. Clinical data of NPC and rhinitis patients

NPC = narsopharengeal carcinoma. NPC differentiation was classified according to the WHO standard.

Supplemental Figures and Figure Legends

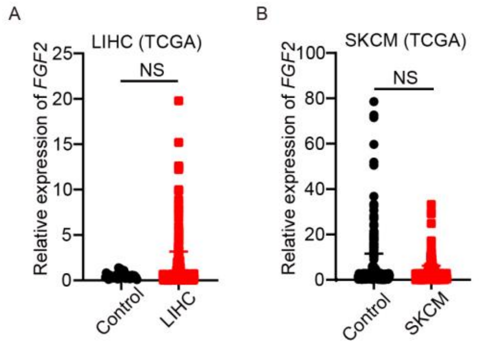

Figure S1. ***FGF2*** **expression in hepatocellular carcinoma and cutaneous melanoma.**

(A) Transcriptomic expression levels of *FGF2* in human LIHC tissues and their adjacent healthy tissues (control, n=160 samples; LIHC, n=369 samples). (B) Transcriptomic expression levels of *FGF2* in human SKCM tissues and their adjacent healthy tissues (control, n=558 samples; SKCM, n=461 samples). NS = not significant. Data presented as mean±SD.

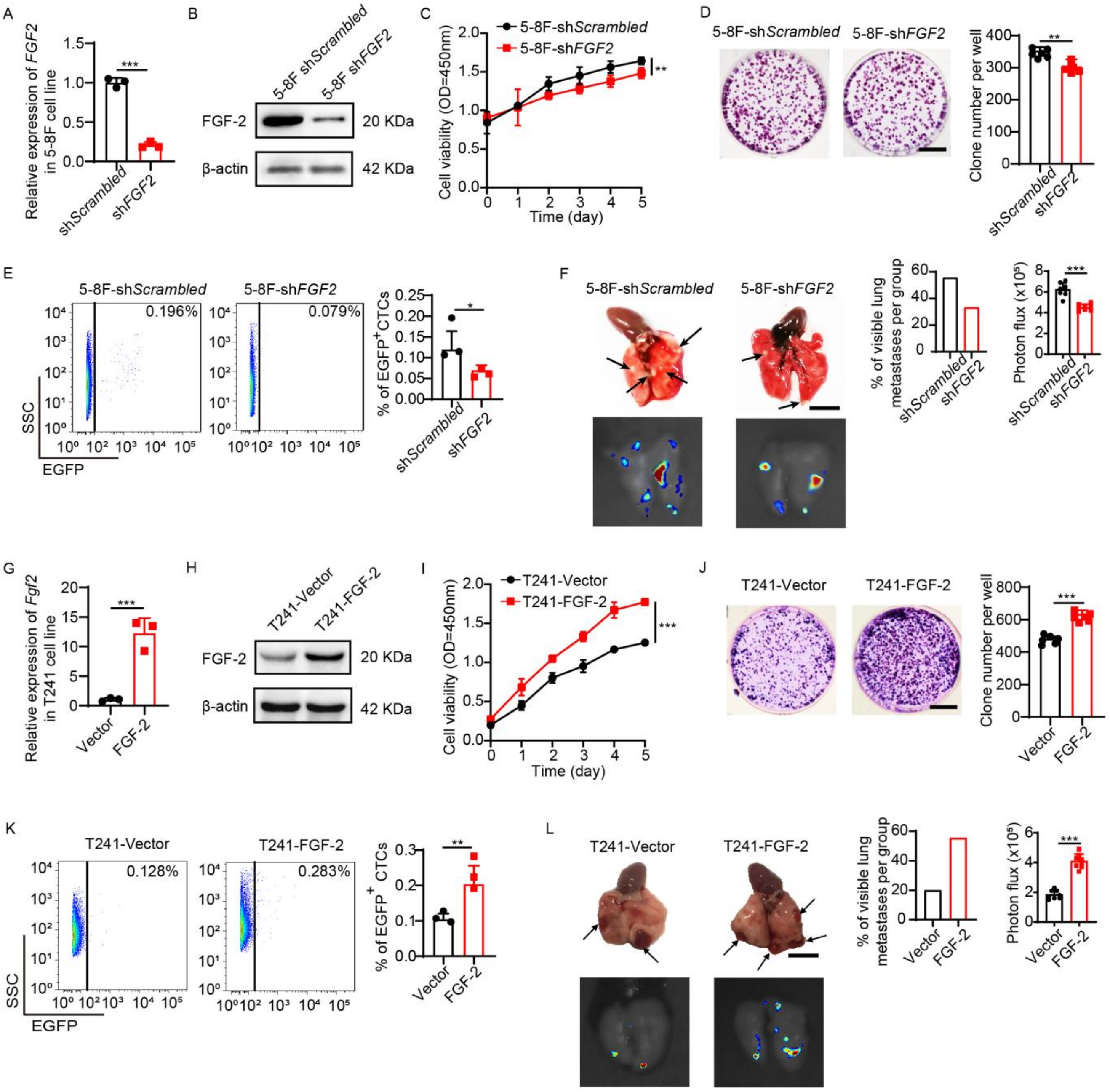

Figure S2. FGF-2 increase tumor cell growth in vitro.

(A and G) QPCR quantification of *FGF2* mRNA levels in sh*Scramble*- and sh*FGF2*-transfected NPC tumor cells and vehicle- and FGF-2-transfected T241 tumor cells (n=3 samples per group). (B and H) FGF-2 upregulation or downregulation in sh*Scramble*- and sh*FGF2*-transfected NPC tumor cells and vehicle- and FGF-2-transfected T241 tumor cells. β-actin marks the loading level in each lane. These experiments were repeated twice. (C and I) Growth rates (n=8 samples per group) and clone formation (n=6 samples per group) (D and J) of

sh*Scramble*- and sh*FGF2*-transfected NPC tumor cells and vehicle- and FGF-2-transfected T241 tumor cells in vitro. (E and K) Blood samples from 5-8F-sh*Scrambled* or 5-8F-sh*FGF2* tumor-bearing mice (E) and from vector and FGF-2 overexpressing tumor-bearing mice (K) were FACS analysed for $EGFP^+$ signals. Quantification of $EGFP^+$ circulating tumor cells in blood samples (n=3 samples randomly chosen from 8 mice per group). (F and L) Representative micrographs of lungs from 5-8F-sh*Scrambled* or 5-8F-sh*FGF2* tumor-bearing mice (F) and from vector or FGF-2 overexpressing tumor-bearing mice (L). Arrows indicate visible metastatic nodules. Scale bar=0.5 cm. $EGFP^+$ metastatic signals in the lung. Quantification of visible lung metastasis (n=8 mice per group). $^{**}p<0.01$; $^{***}p<0.001$. Data presented as mean±SD.

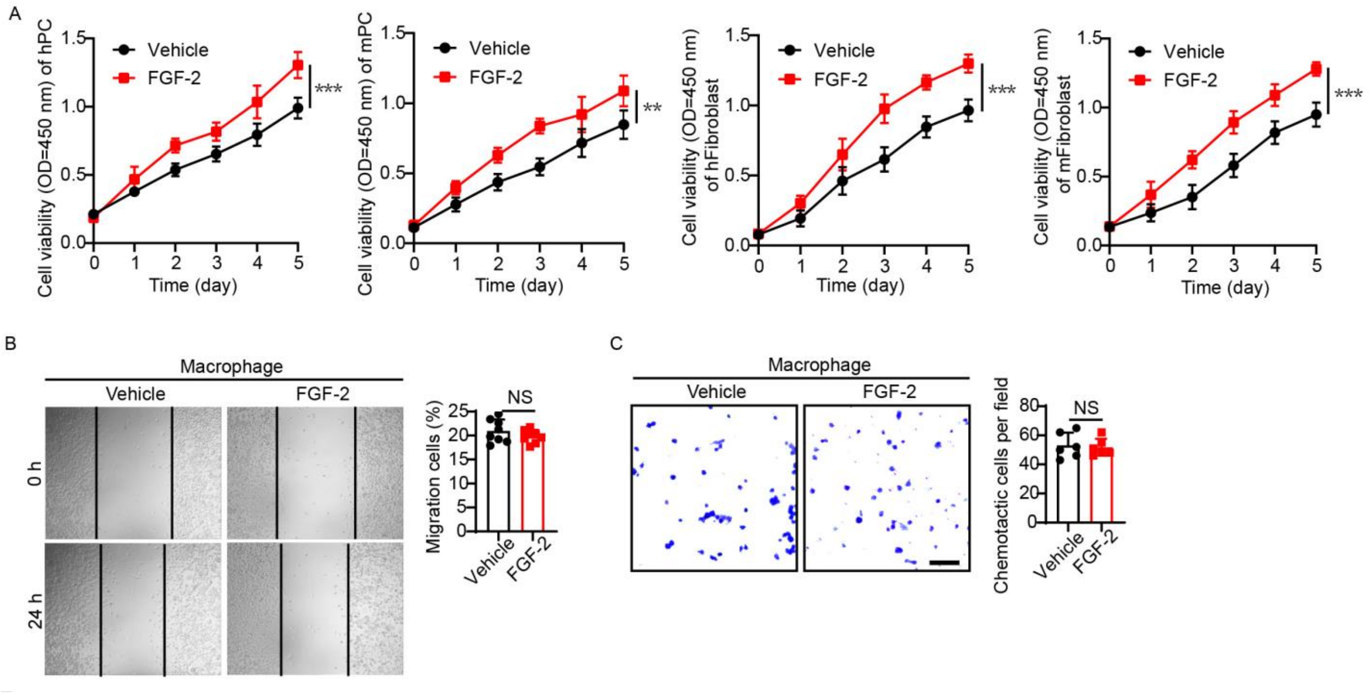

Figure S3. FGF-2 promotes pericyte and fibroblast proliferation but not macrophage migration.

(A) Growth rates of vehicle- or FGF-2-stimulated human and mouse pericytes/fibroblasts in vitro. (B and C) Cell migration (n=8 samples per group) and chemotactic ability (n=6 samples per group) of vehicle- or FGF-2-stimulated macrophages. **p<0.01; ***p<0.001. NS=not significant. Data presented as mean±SD.

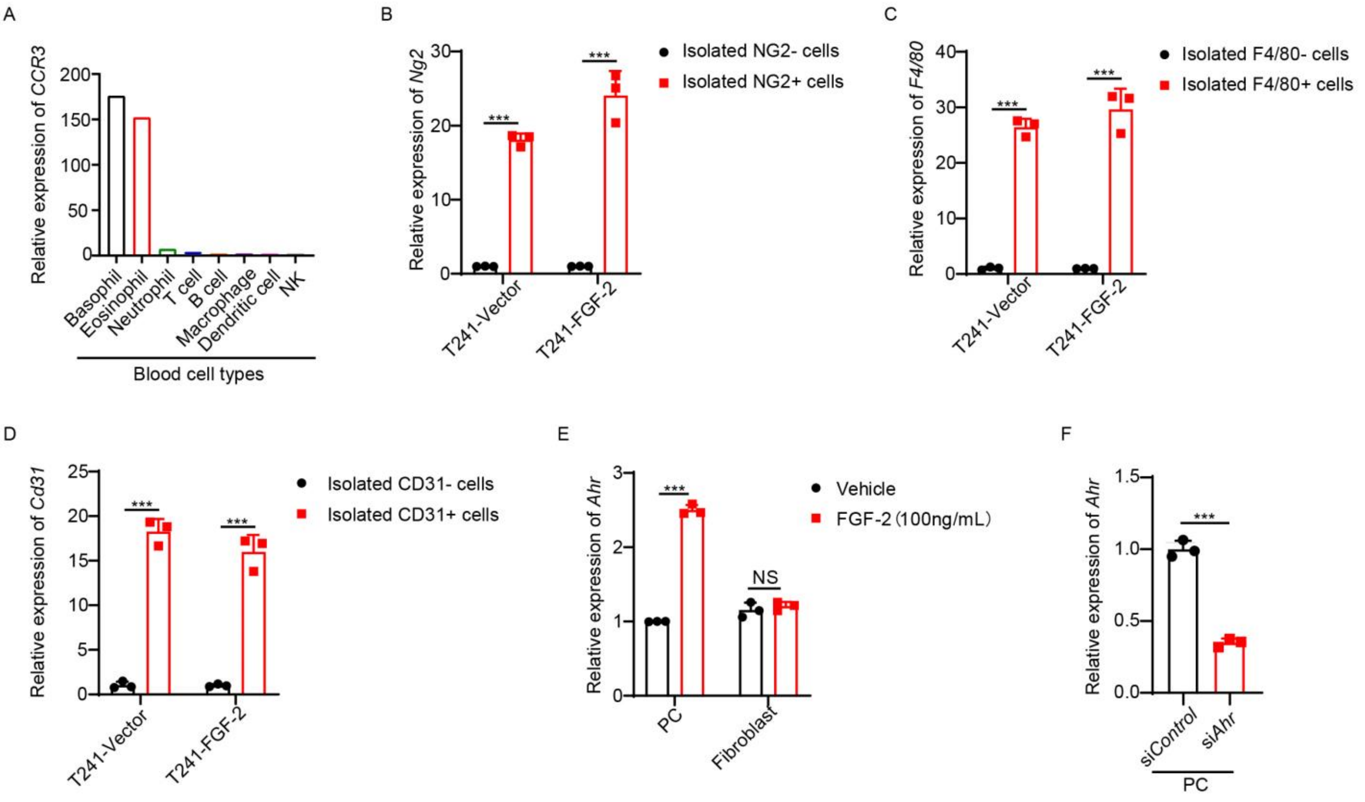

Figure S4. Cell isolation efficiency and AHR expression in FGFR positive cells.

(A) CCR3 expression in various human immune cell types. Data were downloaded from the Human Protein Atlas. (B-D) QPCR quantification of *Ng2*, *F4/80*, *CD31* mRNA levels in isolated cells from vehicle- and FGF-2-transfected T241 tumors (n=3 samples per group). (E) Expression levels of *Ahr* in vehicle- and FGF-2-stimulated isolated primary pericytes and MS5 fibroblasts (n=3 samples per group). (F) QPCR quantification of *Ahr* mRNA levels in isolated primary pericytes (n=3 samples per group). ***p<0.001. NS=not significant. Data presented as mean±SD.

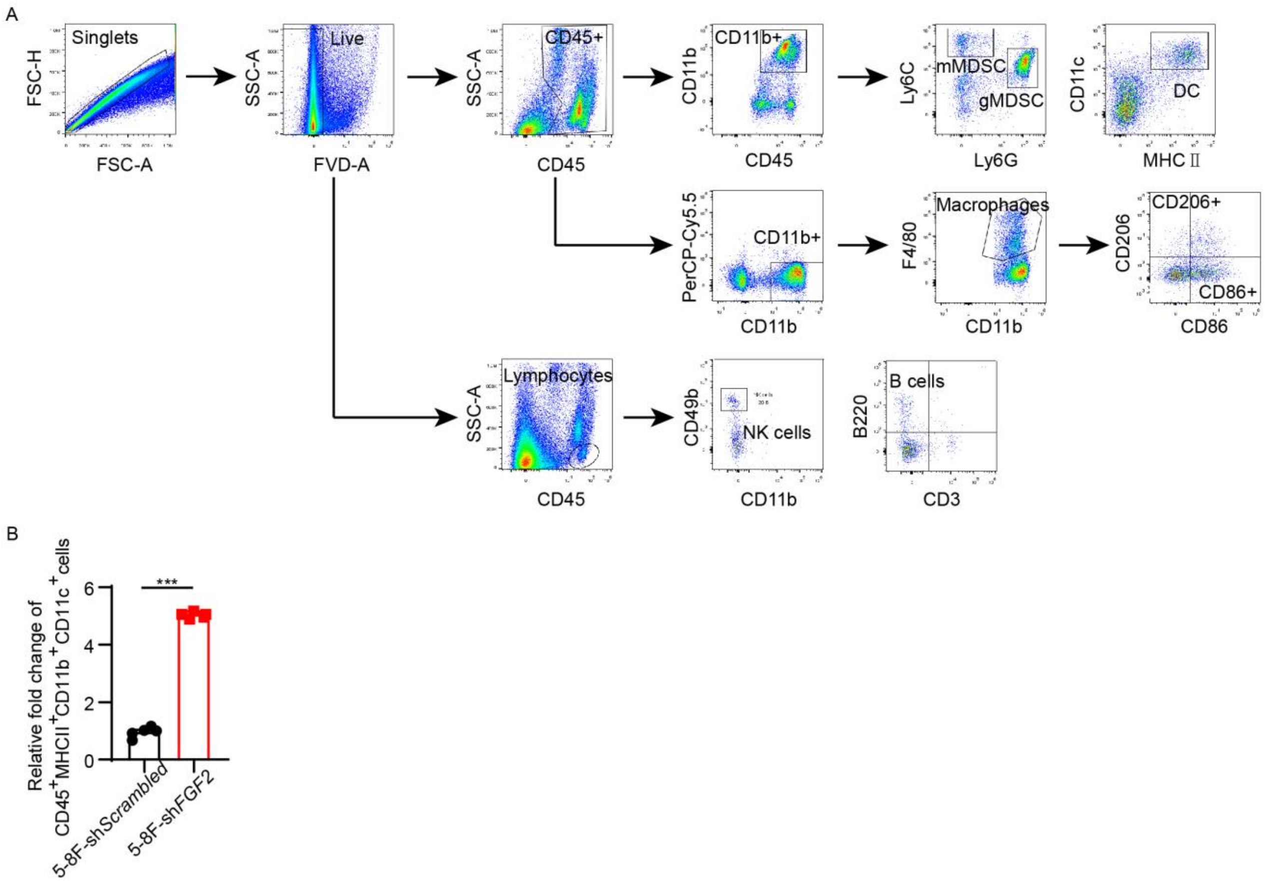

Figure S5. FGF-2 knockdown increases DC infiltration in TME.

(A) Representative graph of FACS analysis showing the gating strategy to identify various infiltrated immune cells in the NPC microenvironment (n=5 samples per group). (B) Quantification of $CD45^+$ $MHCII^+$ $CD11b^+$ $CD11c^+$ dendritic cell population (n=5 mice per group). ***$p<0.001$. Data presented as mean±SD.

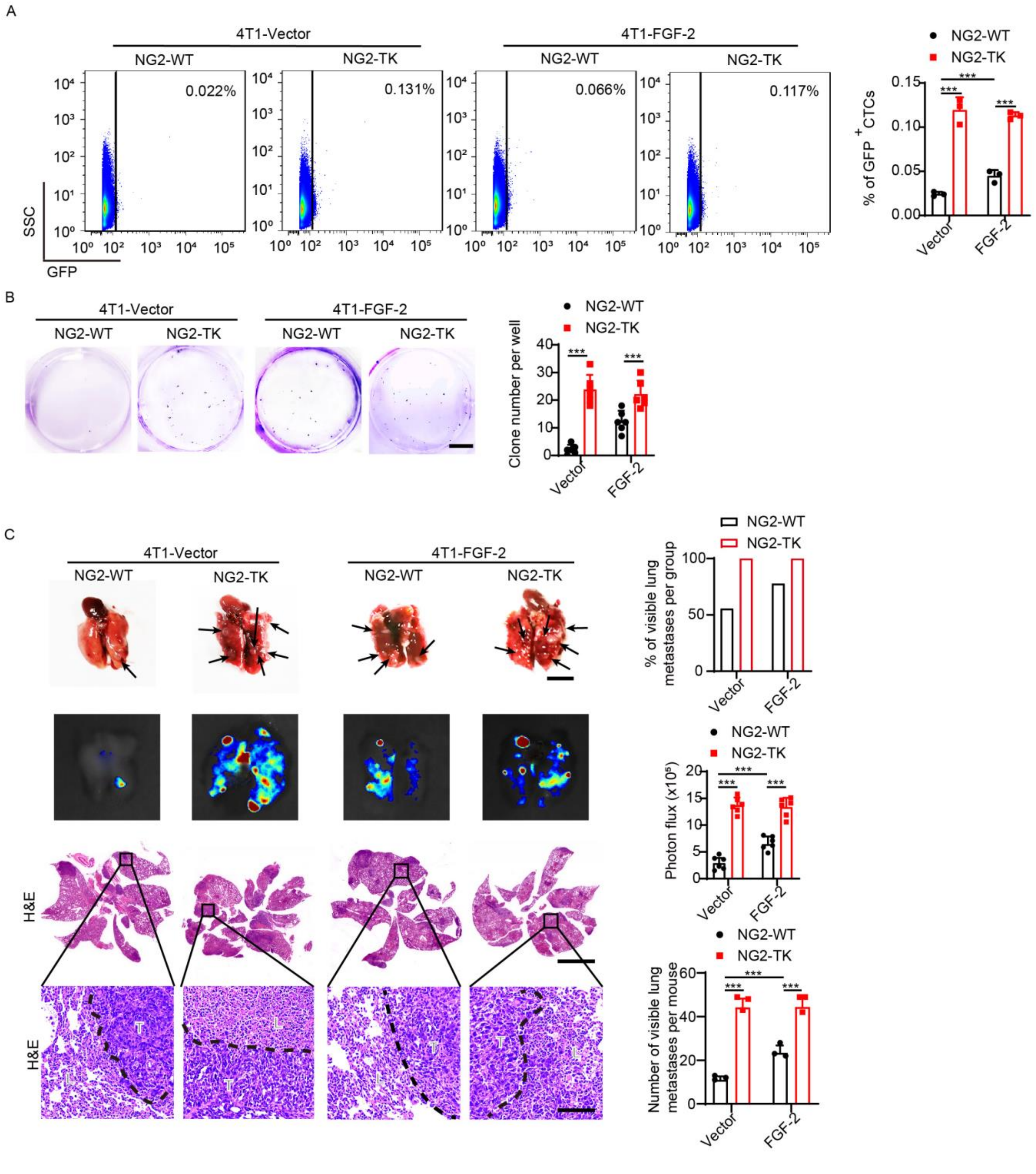

Figure S6. Genetic depletion of pericytes promotes metastasis.

(A) Blood samples from 4T1-Vector or 4T1-FGF-2 tumor-bearing wild type and NG2-TK mice were FACS analysed for $EGFP^+$ signals. Quantification of $EGFP^+$ circulating tumor cells in blood samples (n=3 samples randomly chosen from 6 mice per group). (B) Micrographs of representative cell culture dishes after incubation with blood samples from 4T1-Vector or 4T1-

FGF-2 tumor-bearing wild type and NG2-TK mice. Blue signal indicates the crystal violet-positive tumor colonies. Scale bar=1 cm. (C) Representative micrographs of lungs from 4T1-Vector or 4T1-FGF-2 tumor-bearing wild type and NG2-TK mice. Arrows indicate visible metastatic nodules. Scale bar=0.5 cm. $EGFP^+$ metastatic signals in the lung. Quantification of visible lung metastasis (n=6 mice per group). H&E staining in the lung from 4T1-Vector or 4T1-FGF-2 tumor-bearing wild type and NG2-TK mice. Scale bar in upper panel=3 mm, scale bar in lower panel=100 μm. Quantification of total microscopic lung metastases and various size of metastases (n=3 samples randomly chosen from 6 mice per group). ***$p<0.001$. Data presented as mean±SD.

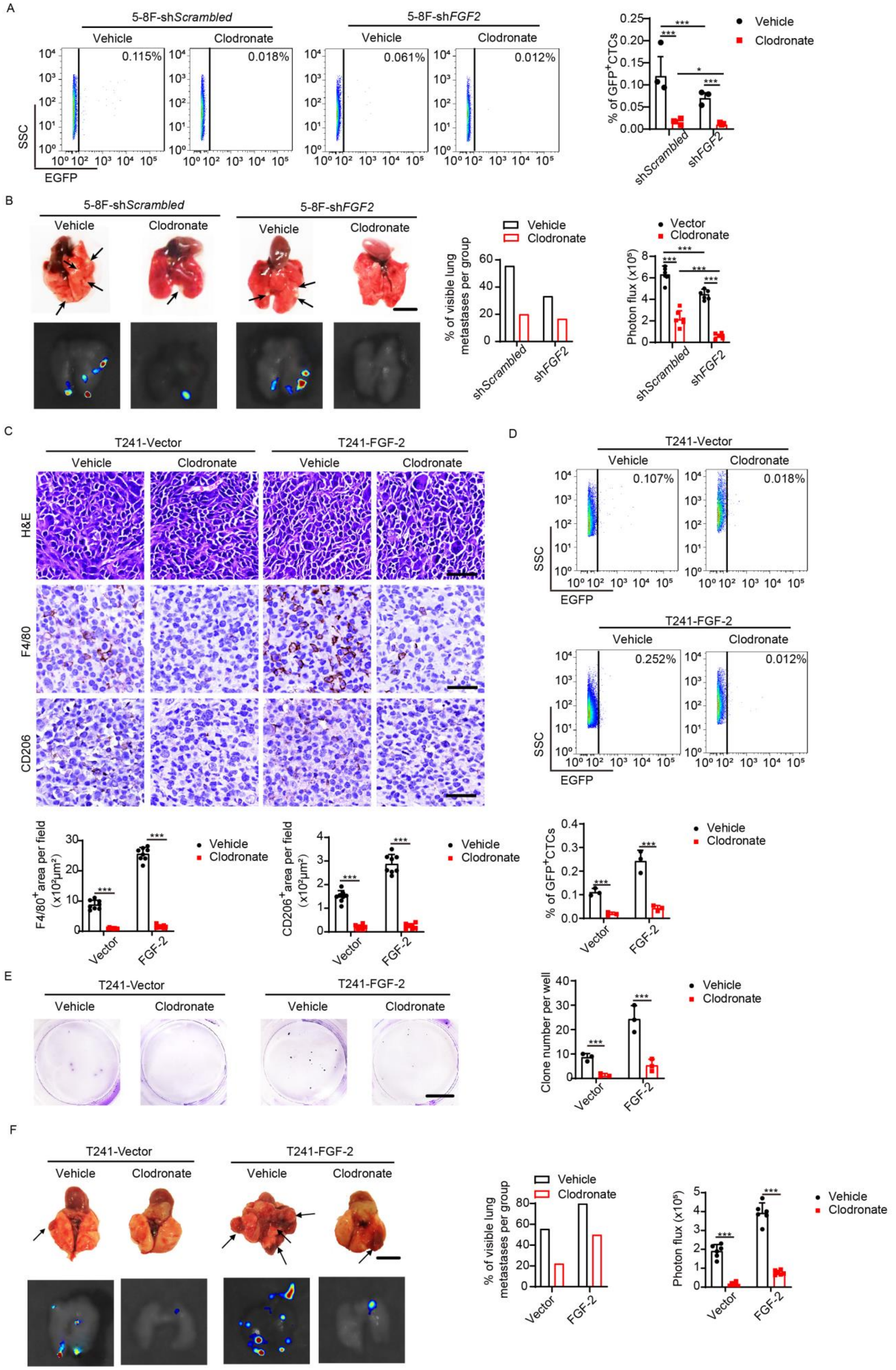
A
5-8F-shScrambled
5-8F-shFGF2
Vehicle
Clodronate
0.115%
0.018%
0.061%
0.012%
SSC
EGFP
% of GFP+CTCs
Vehicle
Clodronate
shScrambled
shFGF2
B
% of visible lung metastases per group
Photon flux (x10⁵)
Vector
C
T241-Vector
T241-FGF-2
H&E
F4/80
CD206
F4/80+area per field (x10²μm²)
CD206+area per field (x10²μm²)
D
0.107%
0.018%
0.252%
0.012%
E
Clone number per well
F

Figure S7. Pharmacological TAM depletion diminishes FGF-2-induced metastasis.

(A) Blood samples from 5-8F-sh*Scrambled* or 5-8F-sh*FGF2* tumor-bearing mice receiving vehicle or clodronate liposomes were FACS analysed for $EGFP^+$ signals. Quantification of $EGFP^+$ circulating tumor cells in blood samples (n=3 samples randomly chosen from 6 mice per group). (B) Representative micrographs of lungs from 5-8F-sh*Scrambled* or 5-8F-sh*FGF2* tumor-bearing mice receiving vehicle or clodronate liposomes. Arrows indicate visible metastatic nodules. Scale bar=0.5 cm. $EGFP^+$ metastatic signals in the lung. Quantification of visible lung metastasis (n=6 mice per group). (C) Micrographs of H&E and immunohistochemistry staining with F4/80 (brown), CD206 (brown) in T241-Vector or T241-FGF-2 tumors implanted in clodronate-treated and non-treated mice. Scale bar=50 μm. Quantification of $F4/80^+$ and $CD206^+$ signals (n=8 random fields per group). (D) Blood samples from T241-Vector or T241-FGF-2 tumor-bearing mice receiving vehicle or clodronate liposomes were FACS analysed for $EGFP^+$ signals. Quantification of $EGFP^+$ circulating tumor cells in blood samples (n=3 samples randomly chosen from 6 mice per group). (E) Micrographs of representative cell culture dishes after incubation with blood samples from T241-Vector or T241-FGF-2 tumor-bearing mice receiving vehicle or clodronate liposomes. Blue signal indicates the crystal violet-positive tumor colonies. Scale bar=1 cm. (F) $EGFP^+$ metastatic signals in the lung. Quantification of visible lung metastasis (n=6 mice per group). *p<0.05; ***p<0.001. Data presented as mean±SD.
